# The transcription factor Rpn4 activates its own transcription and induces efflux pump expression to confer fluconazole resistance in *Candida auris*

**DOI:** 10.1128/mbio.02688-23

**Published:** 2023-11-28

**Authors:** Eve W. L. Chow, Yabing Song, Jinxin Chen, Xiaoli Xu, Jianbin Wang, Kun Chen, Jiaxin Gao, Yue Wang

**Affiliations:** 1 Infectious Diseases Labs, Agency for Science, Technology and Research, Singapore, Singapore; 2 School of Life Sciences, Tsinghua University, Beijing, China; 3 State Key Laboratory of Mycology, Institute of Microbiology, Chinese Academy of Sciences, Beijing, China; 4 Beijing Frontier Research Center for Biological Structure, Tsinghua University, Beijing, China; 5 Translational Medical Center for Stem Cell Therapy, Institute for Regenerative Medicine, Shanghai East Hospital, School of Life Sciences and Technology, Tongji University, Shanghai, China; 6 Shanghai Key Laboratory of Signaling and Disease Research, Frontier Science Center for Stem Cell Research, School of Life Sciences and Technology, Tongji University, Shanghai, China; 7 Department of Biochemistry, Yong Loo Lin School of Medicine, National University of Singapore, Singapore, Singapore; Tel Aviv University, Tel Aviv, Israel

**Keywords:** *Candida auris*, antifungal resistance, Rpn4 transcription factor, efflux pumps, transposon-mediated mutagenesis

## Abstract

The emergence and nosocomial spread of the multidrug-resistant *Candida auris* pose a severe public health threat. Most *C. auris* clinical isolates are fluconazole resistant. In this study, we conduct transposon-mediated screens to profile genes whose inactivation causes fluconazole resistance in *C. auris*. We discover that mutation of genes encoding the Ubr2/Mub1 ubiquitin-ligase complex results in high fluconazole resistance by stabilizing the transcription activator, Rpn4. Global transcriptomic analysis, quantitative PCR, and combinatorial gene deletion reveal that Rpn4 causes fluconazole resistance by upregulating four efflux pump genes, *SNQ21*, *SNQ22*, *MDR1*, and *CDR1*, increasing efflux activity of the cell. Rpn4 autoactivates its own expression by binding to a PACE element in its promoter, forming a positive autoregulatory loop. Rpn4 also promotes *CDR1* expression by binding to a PACE element in the *CDR1* promoter. Furthermore, using the published genome sequence data of 304 clinical isolates, we identified a *UBR2* A316T mutation in isolates from clades I and III. Although complementation of the *ubr2*Δ mutant with the *UBR2*^A316T^ allele resulted in increased susceptibility to fluconazole, the *UBR2*^A316T^ mutant was still more resistant to fluconazole compared to wild type, suggesting that acquisition of the mutation is sufficient to confer an increase in fluconazole resistance. In conclusion, this study identifies Rpn4 as a critical transcription factor that regulates fluconazole resistance via the Rpn4-efflux pump axis in *C. auris*.

**IMPORTANCE:**

*Candida auris* is a recently emerged pathogenic fungus of grave concern globally due to its resistance to conventional antifungals. This study takes a whole-genome approach to explore how *C. auris* overcomes growth inhibition imposed by the common antifungal drug fluconazole. We focused on gene disruptions caused by a “jumping genetic element” called transposon, leading to fluconazole resistance. We identified mutations in two genes, each encoding a component of the Ubr2/Mub1 ubiquitin-ligase complex, which marks the transcription regulator Rpn4 for degradation. When either protein is absent, stable Rpn4 accumulates in the cell. We found that Rpn4 activates the expression of itself as well as the main drug efflux pump gene *CDR1* by binding to a PACE element in the promoter. Furthermore, we identified an amino acid change in Ubr2 in many resistant clinical isolates, contributing to Rpn4 stabilization and increased fluconazole resistance.

## INTRODUCTION

The recent emergence of the fungal pathogen *Candida auris* has caused grave concerns globally due to its rapid spread, multidrug resistance, stress tolerance, and frequent outbreaks associated with high mortality (1–3). Since its first isolation in Japan in 2009, *C. auris* has been reported in more than 40 countries across six continents (4–6). Currently, the majority of *C. auris* clinical isolates fall into four geographic clades, which emerged nearly simultaneously across the world (4, 5). Whole-genome sequencing has revealed hundreds of thousands of single nucleotide polymorphisms (SNPs) between isolates of different clades, although genetic diversity is low within each clade (7). Unlike other members of the *Candida* family, *C. auris* presents a set of unique clinically important characteristics. An alarming trait of *C. auris* is the exceedingly high percentage of drug-resistant isolates, including resistance to all three classes of antifungals approved for treating invasive fungal diseases, severely limiting treatment options (7, 8). Also, this fungus can withstand a range of harsh environmental stresses, including high temperature (9, 10), high osmotic pressure (9, 10), and disinfectants commonly used to kill germs (11), rendering its eradication challenging, leading to its persistence and person-to-person transmission in hospitals and healthcare facilities (2, 3, 12). In addition, many clinical isolates grow in aggregates of various sizes, resulting in resistance to physical disruption, reduced susceptibility to antifungal therapies, and evasion from the host immune system (13, 14). As of 2019, the U.S. Centers for Disease Control and Prevention has categorized *C. auris* as the second nationally notifiable fungal disease after coccidioidomycosis and issued multiple warnings (15). Despite the medical importance of *C. auris*, our knowledge about its biology and infection-related traits remains limited, underscoring a pressing need to conduct mechanistic investigations of this new pathogen to develop effective preventive and therapeutic strategies.

The worldwide prevalence of antifungal resistance seriously threatens public health and presents substantial clinical challenges (16). The problem is exacerbated by the patient-to-patient transmission of *C. auris* through direct contact or exposure to contaminated surfaces and fomites when shed from infected or colonized patient (3, 17, 18). Over time, treatment and lateral transmission can quickly result in drug-resistant *C. auris*, like what happens in bacterial pathogens (19). Indeed, antifungal resistance, especially against fluconazole, is strikingly common among *C. auris* isolates. Different studies have reported 80%–100% of *C. auris* clinical isolates with increased resistance to fluconazole, the most widely prescribed azole antifungal to treat systemic *Candida* infections (4, 7) . Although mechanisms of fluconazole resistance are highly variable and often clade-specific, several general resistance mechanisms have been identified in *C. auris*, such as mutation or duplication of the drug target gene, *ERG11* (4, 7, 20–24), and transcriptional upregulation of efflux transporters, Cdr1 and Mdr1 (23, 25–29). In *Candida albicans*, overexpression of *CDR1* and *MDR1* is controlled by the transcription factors Tac1 (30, 31) and Mrr1 (32, 33), respectively. Genome sequence analyses of fluconazole-resistant *C. auris* clinical isolates have revealed an association of several mutations in *TAC1b*, one of two *C. auris* homologs of *C. albicans TAC1*, with fluconazole resistance, including S611P, A640V, A657V, and F862_N866del (22, 34). Two mutations, R495G and F214S, were identified in *TAC1b* in five fluconazole-resistant strains that evolved independently in the presence of fluconazole *in vitro* with increased expression of *CDR1* (34). However, whether *C. auris TAC1* regulates *CDR1* overexpression remains unclear due to the lack of direct analyses of the impact of *TAC1b* mutations on *CDR1* expression. Furthermore, another study showed that the loss of either *TAC1a* or *TAC1b* had no significant effect on the *CDR1* transcript level (35), strongly suggesting the presence of yet unknown regulators of *CDR1* expression and fluconazole resistance in *C. auris*.

In this study, we conducted genome-wide genetic screens using the *piggyBac* (*PB*)-transposon mutagenesis system to identify genes whose inactivation causes fluconazole resistance in *C. auris*. We found that mutation of the E3 ligase Ubr2 or its adaptor Mub1 resulted in high fluconazole resistance by stabilizing the transcription activator, Rpn4, and thus increasing its cellular level. Global transcriptomic analysis, quantitative PCR (qPCR), and combinatorial gene deletion revealed that in *ubr2*Δ and *mub1*Δ mutants, increased cellular levels of Rpn4 upregulate the expression of four efflux pump genes, most notably *CDR1*, leading to fluconazole resistance. Moreover, Rpn4 autoactivates its own expression by binding to a PACE element in its promoter and further directly upregulates *CDR1* expression by the same mechanism. Finally, we highlighted the clinical relevance of this Ubr2/Mub1-Rpn4-efflux pump signaling pathway by assessing the influence of a clinically derived *UBR2* mutation, A316T, on fluconazole susceptibility. This study identifies Rpn4 as a critical transcription factor that directly activates efflux pump expression to confer fluconazole resistance in *C. auris*.

## RESULTS

### Identification of *RPN4* as a key determinant of fluconazole resistance in *C. auris*

**Fig 1:**
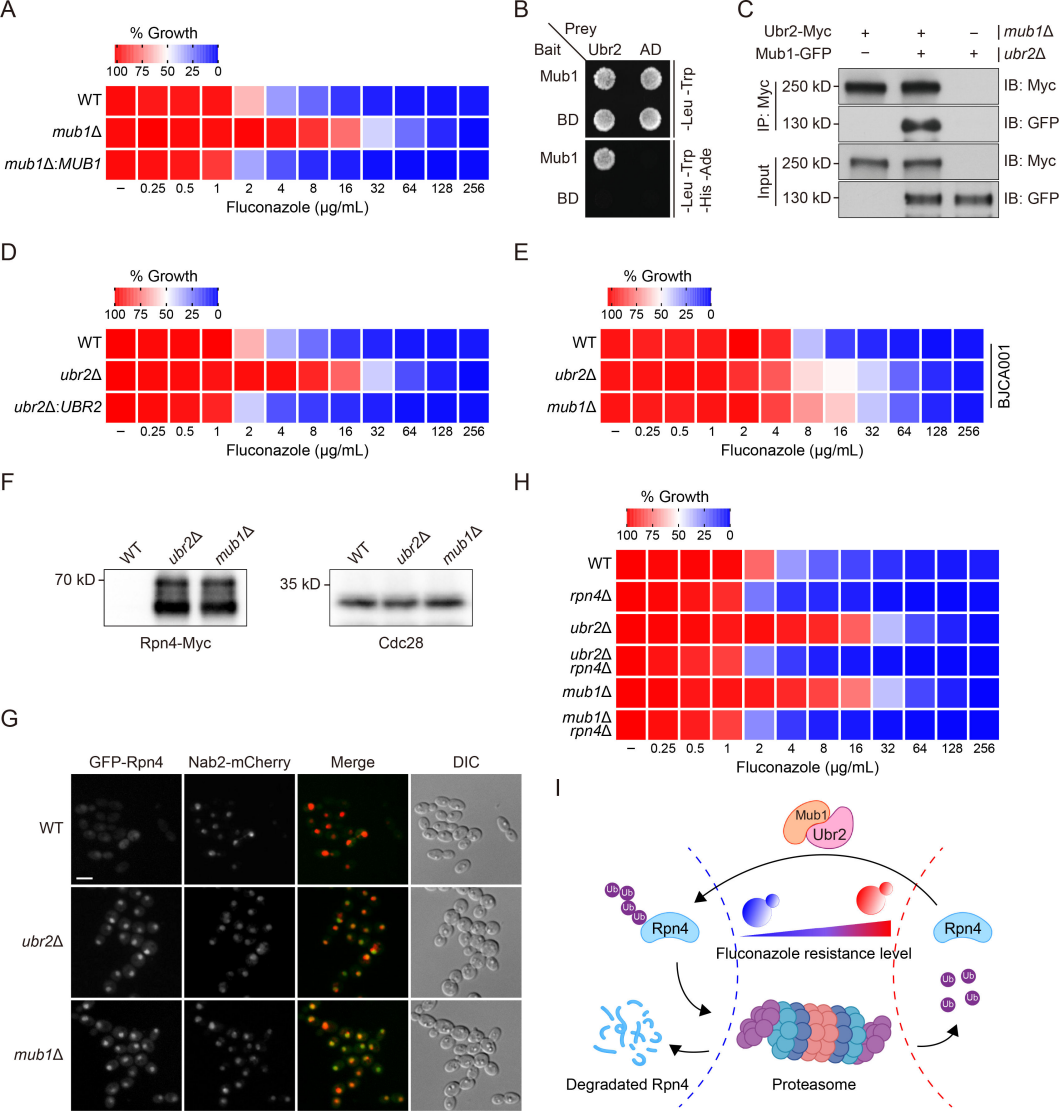
*RPN4* is a key determinant of fluconazole resistance in *C. auris*. (**A**) Fluconazole susceptibility assay. WT, *mub1*Δ, and *mub1*Δ:*MUB1* cells were inoculated into YPD containing twofold serially diluted fluconazole and incubated at 30°C for 72 h. Growth was determined by taking the optical density at 600 nm (OD_600_) and expressed as the relative growth with the no-drug well of each strain set as 100%. Data are representative of three technical replicates. (**B**) Mub1 interacts with Ubr2 in the yeast two-hybrid assay. The combinations of BD-Mub1 with the empty prey vector (AD), AD-Ubr2 with the empty bait vector (BD), and AD with BD served as negative controls. (**C**) Co-immunoprecipitation (Co-IP) of Ubr2 with Mub1. *ubr2*Δ cells were used to prevent the degradation of Mub1-green fluorescent protein (GFP). Ubr2-Myc was immunoprecipitated, and Co-IP of Mub1-GFP was analyzed by Western blotting. IB, immunoblotting. (**D**) Fluconazole susceptibility assays for WT, *ubr2*Δ, and *ubr2*Δ:*UBR2* cells were performed as described for panel A. Fluconazole was applied as a twofold dilution series. Growth was measured and normalized to no-drug control. Data are representative of three technical replicates. (**E**) Fluconazole susceptibility assays for *ubr2*Δ and *mub1*Δ mutants generated in BJCA001 (clade I) were performed as described for panel A. Fluconazole was applied as a twofold dilution series. Growth was measured and normalized to no-drug control. Data are representative of three technical replicates. (**F**) The protein level of endogenously expressed Rpn4-Myc in WT, *ubr2*Δ, and *mub1*Δ cells. Cdc28 served as loading control. (**G**) Subcellular localization of GFP-Rpn4 expressed in WT, *ubr2*Δ, and *mub1*Δ cells. Nab2-mCherry served as a nuclear marker. Scale bar, 5 µm. DIC, differential interference contrast. (**H**) Fluconazole susceptibility assays for the indicated strains were performed as described for panel A. Fluconazole was applied as a twofold dilution series. Growth was measured and normalized to no-drug control. Data are representative of three technical replicates. (**I**) Illustration of the results described for this figure. *UBR2* and *MUB1* are critical regulators of *C. auris* fluconazole susceptibility and deletion of either one leads to *RPN4*-dependent fluconazole resistance.

To investigate the mechanisms of fluconazole resistance in *C. auris*, we utilized *PB* transposon*-*mediated mutagenesis to generate an insertional mutant library as described previously (36) and conducted a genetic screen on yeast peptone dextrose (YPD) plates containing 60 µg/mL fluconazole to isolate resistant mutants (Fig. S1A). We initially obtained 12 fluconazole-resistant mutants. However, only four clones were confirmed as true fluconazole-resistant mutants using a dose-response assay based on a twofold serial dilution of fluconazole. A mutant (CauFR4) showing the highest fluconazole resistance among several candidates was chosen for further analysis (Fig. S1B). Inverse PCR and sequencing analysis of this mutant identified a *PB* insertion at a TTAA site (PEKT02000007: 2,736,602–2,736,605) within the open reading frame of *003899*, a homolog of *Saccharomyces cerevisiae MUB1* (Fig. S1C). Deleting *MUB1* in the wild-type (WT) *C. auris* strain CBS10913 (clade II) recapitulated the fluconazole-resistant phenotype (Fig. 1A). Complementing *mub1*Δ cells with the WT *MUB1* (*mub1*Δ:*MUB1*) restored the WT susceptibility level (Fig. 1A), indicating that *MUB1* has a role in regulating fluconazole susceptibility in *C. auris*.

In *S. cerevisiae*, *MUB1* encodes an E2/E3 binding protein that interacts with the E3 ubiquitin-protein ligase Ubr2 (37). Next, we conducted two experiments to determine whether Mub1 forms a complex with Ubr2 in *C. auris*. First, a yeast two-hybrid (Y2H) assay, with Mub1 as the bait and Ubr2 as the prey, indicated a physical interaction between the two proteins (Fig. 1B). Second, we sought to confirm the interaction *in vivo* by co-immunoprecipitation (Co-IP). Although we successfully integrated *MUB1*-GFP at the correct genomic locus, its protein expression could be detected only in *ubr2*Δ cells, suggesting that Mub1 is rapidly degraded in an Ubr2-dependent manner in the WT background (Fig. S2). To overcome this problem, we expressed Ubr2-Myc and Mub1-GFP in *mub1*Δ and *ubr2*Δ cells, respectively, and then combined cell extracts from these two strains for Co-IP. Using this method, we detected the co-precipitation of Mub1-GFP with Ubr2-Myc (Fig. 1C).

Like the *mub1*Δ mutant, *ubr2*Δ cells showed high fluconazole resistance, which was restored to the WT level by reintroducing a copy of WT *UBR2* (*ubr2*Δ:*UBR2*) (Fig. 1D). In addition, deleting either *UBR2* or *MUB1* in the BJCA001 strain of clade I also caused increased fluconazole resistance, albeit to a lesser extent compared to the same deletion in the CBS10913 (Fig. 1E), which supports the conservation of Mub1/Ubr2’s role across different clades. The difference in the magnitude in fluconazole resistance resulting from *UBR2* and *MUB1* deletions in the two different strains is likely due to the extensive genetic diversity between these two clades. For example, isolates from different clades have copy number variations of genes potentially playing a role in host stresses or antifungal drug adaptation (4). Also, BJCA001 contains more copy numbers of the Zorro3 retrotransposon, particularly those inserted close to antifungal resistance-associated genes, which may affect the expression of these genes and thus regulate antifungal susceptibility (38). Nevertheless, the results demonstrate the physical interaction between Mub1 and Ubr2 and their role in regulating *C. auris*’ resistance to fluconazole.

Previous studies in *S. cerevisiae* reported that the Mub1/Ubr2 complex is required for the ubiquitin-dependent degradation of Rpn4, a short-lived transcription activator of proteasome genes, and the loss of either Ubr2 or Mub1 results in elevated Rpn4 levels (37, 39–41). Congruously, Western blotting (WB) detected Rpn4 in *ubr2*Δ and *mub1*Δ cells but not in WT cells (Fig. 1F). Also, fluorescence microscopy detected stronger fluorescent signals of GFP-Rpn4 in *ubr2*Δ and *mub1*Δ cells than in WT cells. Furthermore, GFP-Rpn4 expressed in *ubr2*Δ and *mub1*Δ cells mainly accumulated in the nucleus, colocalizing with a validated nuclear marker, Nab2-mCherry (Fig. 1G). Together, these results indicate that *UBR2* and *MUB1* are responsible for Rpn4 degradation in *C. auris*.

Next, we asked whether the elevated Rpn4 level is responsible for the increased fluconazole resistance of *ubr2*Δ or *mub1*Δ mutants. We measured the synthetic effect of simultaneously deleting *UBR2* or *MUB1* with *RPN4* on fluconazole susceptibility. We found that deletion of *RPN4* in *ubr2*Δ or *mub1*Δ cells reduced fluconazole resistance to a similar level displayed by *rpn4*Δ cells (Fig. 1H). The above results unambiguously demonstrate that *UBR2* and *MUB1* are critical regulators of fluconazole susceptibility in *C. auris* and deletion of either one leads to *RPN4*-dependent fluconazole resistance (Fig. 1I).

### Rpn4 causes fluconazole resistance by transcriptionally activating efflux genes in *C. auris*

**Fig 2:**
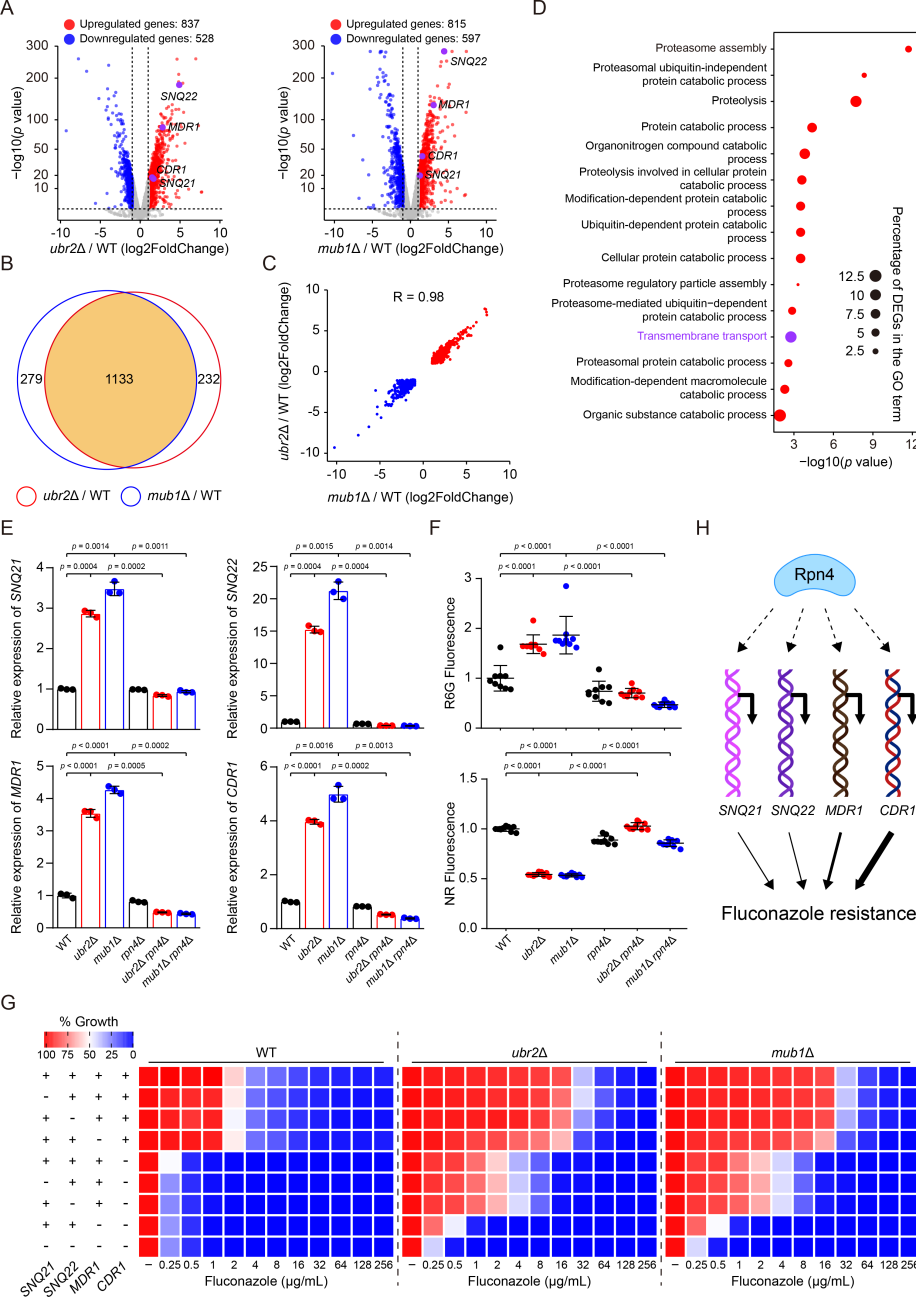
Rpn4 causes *C. auris* fluconazole resistance by overexpressing efflux genes, particularly *CDR1*. (**A**) The volcano plots show DEGs in *ubr2*Δ and *mub1*Δ cells. Numbers of upregulated and downregulated genes are indicated. Four putative efflux genes, *SNQ21*, *SNQ22*, *MDR1*, and *CDR1*, are highlighted in purple. (**B**) Venn diagram shows the overlapping of DEGs in *ubr2*Δ and *mub1*Δ cells. Numbers of unique and common genes are indicated. (**C**) Correlation of the fold change of the overlapped DEGs between *ubr2*Δ and *mub1*Δ cells. (**D**) GO analysis of the overlapped DEGs between *ubr2*Δ and *mub1*Δ cells. The dot plot shows the top 15 upregulated GO terms of biological processes. (**E**) qPCR analysis of *SNQ21*, *SNQ22*, *MDR1*, and *CDR1* expression in the indicated strains. Transcript levels were normalized to *GPD1*. The transcript level of each gene in WT cells was set to 1. Error bars, SD from the mean of three independent experiments. Significance was measured using two-tailed unpaired Student’s *t*-test. (**F**) Efflux activities in the indicated strains were measured using rhodamine 6G (R6G) and Nile Red (NR) as efflux substrates. Cells with strong efflux activity yield higher supernatant R6G fluorescence but less intracellular NR fluorescence. The fluorescent signal produced from WT cells was set to 1. Error bars, SD from the mean of nine technical replicates. Significance was measured using two-tailed unpaired Student’s *t*-test. (**G**) Fluconazole susceptibility assays for the indicated strains were performed as described in Fig. 1A. Fluconazole was applied as a twofold dilution series. Growth was measured and normalized to no-drug control. Data are representative of three technical replicates. (**H**) Illustration of the results described in Fig. 2. Rpn4 causes fluconazole resistance by upregulating the expression of four putative efflux genes, especially *CDR1*, in *C. auris*. The breadth of solid arrow indicates the comparative contributions of each efflux pump to fluconazole resistance.

Recently, two studies have indicated Rpn4’s involvement in fluconazole resistance in *Candida glabrata* and fluconazole tolerance in *C. albicans* through regulating ergosterol biosynthesis genes (42, 43). They proposed the potential conservation of Rpn4’s role in azole susceptibility across fungal species. Considering the distant relationship of *C. auris* with *C. albicans* and *C. glabrata*, we investigated whether Rpn4 alters fluconazole sensitivity in *C. auris* through a similar mechanism or via an as-yet-unknown pathway. We performed RNA sequencing (RNA-Seq) analysis of the total RNA of *ubr2*Δ, *mub1*Δ, and WT cells collected from overnight cultures. As shown in Fig. 2A; Data S1, we detected 1,365 differentially expressed genes (DEGs), 837 upregulated and 528 downregulated, in *ubr2*Δ cells compared with WT cells, and 1,412 DEGs, 815 upregulated and 597 downregulated, in *mub1*Δ cells compared with WT cells. The DEGs identified in the two mutants were highly similar, with a total of 1,133 overlapping genes (Fig. 2B). Furthermore, the fold change of these overlapping genes was highly correlated between the two mutants (*R* = 0.98, Fig. 2C). Together, the results indicated that *ubr2*Δ and *mub1*Δ cells share similar transcriptional profiles. Therefore, we focused our subsequent analysis on the shared DEGs. Gene ontology (GO) term enrichment analysis revealed that most upregulated genes were associated with proteolysis processes (Fig. 2D; Data S2), consistent with previous studies showing *RPN4* as a transcriptional activator of the proteasome genes that regulate proteasome homeostasis (40, 41, 44, 45). However, we did not find the upregulation of any ergosterol biosynthesis genes in *C. auris*. Interestingly, our RNA-Seq data revealed significant downregulation of *ERG3*, *ERG5*, and *ERG11* in both *ubr2*Δ and *mub1*Δ cells (Data S1), indicating that Rpn4 regulates fluconazole resistance by a distinct mechanism in *C. auri*s than in *C. glabrata* and *C. albicans*. Notably, we observed a significant enrichment of genes predicted to be involved in transmembrane transport, including four putative efflux genes, *SNQ21*, *SNQ22*, *MDR1*, and *CDR1* (Fig. 2A and D; Data S2), previously shown to play important roles in drug resistance in various fungal pathogens (23, 46). The data suggested that Rpn4 might confer fluconazole resistance by increasing efflux gene expression in *C. auris*.

To test this idea experimentally, we examined the impact of *RPN4* on the expressions of *SNQ21*, *SNQ22*, *MDR1*, and *CDR1* in *ubr2*Δ, *mub1*Δ, and WT cells by qPCR analysis. We found that the transcript levels of all these four efflux genes are significantly higher in *ubr2*Δ and *mub1*Δ cells than in WT cells and deleting *RPN4* in both mutants reduced their expression to the WT level (Fig. 2E). These results verified that Rpn4 positively regulates the expression of efflux genes in *C. auris*.

Next, we measured efflux pump activities in the same set of strains using two fluorescent efflux pump substrates, rhodamine 6G (R6G) (47) and Nile Red (NR) (48). Both compounds can enter and accumulate in fungal cells and are actively removed by efflux pumps. R6G is a substrate of ABC transporters; after incubation with fungal cells, higher extracellular fluorescence correlates to more robust ABC transporter efflux activity. NR is a substrate of ABC transporters and major facilitator superfamily transporters and only fluoresces in highly hydrophobic environments inside the cell. Thus, cells with higher efflux activity yield less fluorescence. Congruent with the qPCR results, both *ubr2*Δ and *mub1*Δ cells showed increased export of R6G and NR (Fig. 2F). Deletion of *RPN4* in these two mutants reduced R6G and NR efflux activities to levels comparable to that in WT cells (Fig. 2F). This observation is consistent with previous reports of an increase in the transcript level of efflux pump genes leading to elevated efflux activity (34, 49). Together, these results indicate that Rpn4 confers fluconazole resistance by transcriptionally activating efflux transporters in *ubr2∆* and *mub1∆* mutants.

Next, we characterized the relative contributions of each efflux pump by performing fluconazole susceptibility tests with *SNQ21*, *SNQ22*, *MDR1*, or *CDR1* single deletion mutants generated in *ubr2*Δ, *mub1*Δ, and WT strains. Deleting *SNQ21* or *SNQ22* alone did not alter fluconazole susceptibility in any strain (Fig. 2G). Deleting *MDR1* in *ubr2*Δ and *mub1*Δ cells, but not WT cells, caused a slight decrease in fluconazole resistance (Fig. 2G). In stark contrast, deletion of *CDR1* resulted in markedly increased sensitivity to fluconazole across all strains (Fig. 2G). These findings demonstrate that Cdr1 plays a dominant role in fluconazole resistance compared to Snq21, Snq22, and Mdr1. Although single deletion of *SNQ21*, *SNQ22*, or *MDR1* had little effect on fluconazole resistance, we speculated that the presence of *CDR1* could mask their effects on fluconazole resistance. To explore this possibility, we further deleted *SNQ21*, *SNQ22*, or *MDR1* individually or in combination in *ubr2*Δ *cdr1*Δ, *mub1*Δ *cdr1*Δ, and *cdr1*Δ cells and examined their fluconazole susceptibility. Deleting either *SNQ21* or *SNQ22* in any of the three strains did not have a notable effect (Fig. 2G). In contrast, deleting *MDR1* in *ubr2*Δ *cdr1*Δ or *mub1*Δ *cdr1*Δ cells increased fluconazole susceptibility substantially (Fig. 2G). Additionally, quadruple deletions of these transporter genes in *ubr2*Δ and *mub1*Δ cells led to a further increase in fluconazole susceptibility (Fig. 2G), suggesting a collective contribution of these transporters to fluconazole resistance. Previous reports in *S. cerevisiae* and *C. albicans* also suggested a model in which Rpn4 activates proteasome capacity transcriptionally for overcoming drug-induced proteotoxicity, resulting in fluconazole resistance or tolerance (43, 50). Although our RNA-Seq data revealed a positive correlation between proteasome gene expression and Rpn4 abundance, it appears unlikely that Rpn4-dependent regulation of proteasome activity contributes to the fluconazole resistance observed in *ubr2*Δ and *mub1*Δ mutants. This is because deletion of the four efflux pumps simultaneously in *ubr2*Δ and *mub1*Δ cells reduced fluconazole susceptibility to the same extent as such deletion in a WT background (Fig. 2G). Considering all these findings, we conclude that fluconazole resistance caused by Rpn4 results from the transcriptional activation of multiple efflux pump genes, especially *CDR1*, in *C. auris* (Fig. 2H).

### Rpn4 autoactivates its transcription by binding to its own promoter

**Fig 3:**
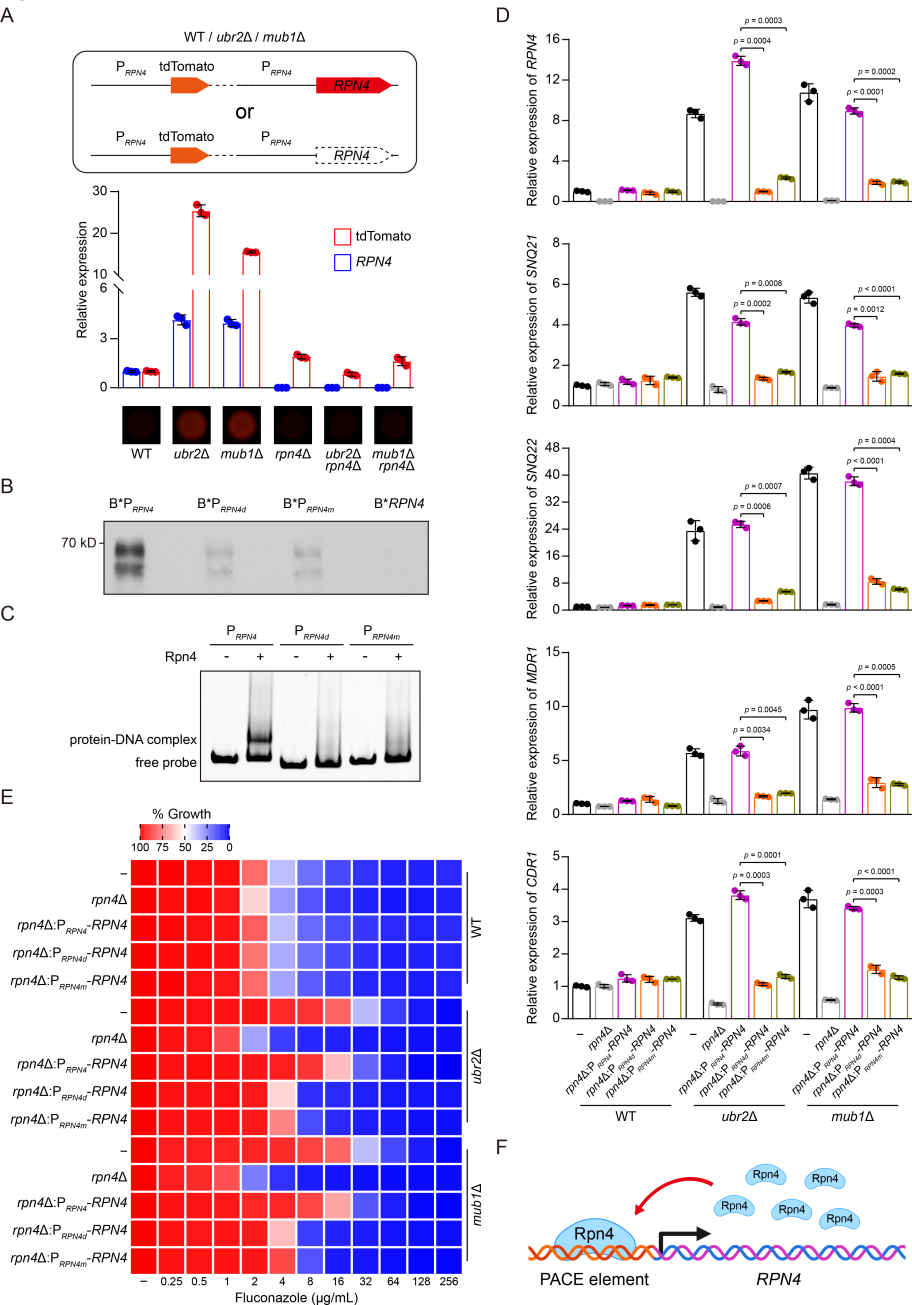
*RPN4* positive autoregulation is essential for efflux genes upregulation and fluconazole resistance in *C. auris*. (**A**) A functional tdTomato protein under the control of the *RPN4* promoter was introduced into WT, *ubr2*Δ, and *mub1*Δ cells in the presence or absence of *RPN4*. The fluorescent tdTomato signals and the transcript levels of tdTomato and *RPN4* were measured by the fluorescence stereomicroscope and qPCR, respectively. Transcript levels were normalized to *GPD1*. The transcript level of each gene in WT cells was set to 1. Error bars, SD from the mean of three independent experiments. Significance was measured using two-tailed unpaired Student’s *t*-test. (**B**) DNA pulldown assay was performed by adhering the biotinylated WT (B*P*_RPN4_*), PACE element-deleted (B*P*_RPN4d_*) or -mutated (B*P*_RPN4m_*) *RPN4* promoter fragments to the streptavidin-conjugated beads, and then incubated the DNA-bound beads with the cell lysates extracted from *ubr2*Δ cells expressing Rpn4-Myc. *ubr2*Δ background was used to prevent the degradation of Rpn4-Myc. The same-length biotinylated *RPN4* coding sequence fragment was included as a negative control (B*RPN4). WB was performed using an anti-Myc antibody. (**C**) Gel mobility shift assay was performed by incubating the WT (P*_RPN4_*), PACE element-deleted (P*_RPN4d_*) or -mutated (P*_RPN4m_*) *RPN4* promoter fragments with the purified recombinant Rpn4 produced in *Escherichia coli*. The formation of protein-DNA complex was analyzed using a 6% nondenaturing polyacrylamide gel with SYBR Green staining. (**D**) qPCR analysis of *RPN4*, *SNQ21*, *SNQ22*, *MDR1*, and *CDR1* expression in the indicated strains. Transcript levels were normalized to *GPD1*. The transcript level of each gene in WT cells was set to 1. Error bars, SD from the mean of three independent experiments. Significance was determined using two-tailed unpaired Student’s *t*-test. (**E**) Fluconazole susceptibility assays for the indicated strains were performed as described in Fig. 1A. Fluconazole was applied as a twofold dilution series. Growth was measured and normalized to no-drug control. Data are representative of three technical replicates. (**F**) Illustration of the results described in Fig. 3. Rpn4 forms a positive feedback loop: it binds to a PACE element in its own promoter to activate its transcription. This autoregulation is essential for efflux genes overexpression and fluconazole resistance in *C. auris*.

The RNA-Seq data revealed a strong upregulation of *RPN4* in *ubr2*Δ and *mub1*Δ cells (Data S1), suggesting that Rpn4 might drive its own expression. To test this possibility, we constructed a transcription reporter by expressing the orange fluorescent protein (tdTomato) under the control of the 1,431 bp upstream sequence of *RPN4* and then integrated this reporter cassette at the *RPN4* locus in *ubr2*Δ, *mub1*Δ, and WT cells in which the endogenous *RPN4* was intact or deleted (Fig. 3A). We detected much higher levels of tdTomato transcripts and tdTomato fluorescence in *ubr2*Δ and *mub1*Δ cells than in WT and *rpn4*Δ cells (Fig. 3A). Consistent with the RNA-Seq data, we also observed an increase of *RPN4* expression in *ubr2*Δ and *mub1*Δ cells compared to WT cells. Together, these results suggest that Rpn4 may positively autoregulate its transcription.

Alignment of Rpn4 amino acid sequences of *C. auris*, *C. albicans*, and *S. cerevisiae* revealed a highly conserved DNA-binding domain despite the overall low similarities (Fig. S3). This finding prompted us to search the *C. auris RPN4* promoter sequence for the proposed consensus binding site of *S. cerevisiae* Rpn4, PACE (5´-GGTGGCAAA-3´) (40). We identified a canonical PACE element at nucleotide (n.t.) −295 to −287 upstream of the START codon. Next, we investigated whether *C. auris* Rpn4 binds to PACE to turn on transcription. We first conducted the DNA pull-down assay. We prepared three biotinylated *RPN4* promoter fragments spanning the region from n.t. −517 to −69 with the PACE motif in the middle. The first fragment was the WT sequence (B*P*_RPN4_*), the second had PACE deleted (B*P*_RPN4d_*), and the third had PACE mutated to 5´-AAGAATAAA-3´ (B*P*_RPN4m_*). We loaded equal amounts of these DNA fragments onto streptavidin-conjugated beads and mixed them with equal amounts of cell lysate extracted from *ubr2*Δ cells expressing Rpn4-Myc for incubation. Proteins pulled down with the beads were probed by WB using an anti-Myc antibody. Figure 3B shows that Rpn4-Myc was readily detected when B*P*_RPN4_* was used as the bait. In comparison, the amount of Rpn4 was markedly reduced when B*P*_RPN4d_* or B*P*_RPN4m_* was used for pulldown. As a negative control, no binding of Rpn4 was detected with a DNA fragment of the same length from the *RPN4* coding sequence (B*RPN4). The results demonstrate that PACE mediates the specific binding of Rpn4 to its own promoter. In another experiment, we purified recombinant Rpn4 expressed in *E. coli* and examined its association with the same set of *RPN4* promoter fragments described above but without biotinylation. After incubating P*_RPN4_* with recombinant Rpn4, we conducted the electrophoretic mobility shift assay. We observed a retarded mobility of P*_RPN4_* after incubating with the recombinant Rpn4, indicating the formation of a protein-DNA complex (Fig. 3C). In contrast, the mobility of P*_RPN4d_* and P*_RPN4m_* was not affected after incubation with the recombinant Rpn4 (Fig. 3C). Together, these results demonstrate direct binding of Rpn4 to its own promoter via the PACE element.

To confirm that the PACE-mediated binding of Rpn4 to its own promoter activates its transcription, we expressed full-length *RPN4* in *ubr2*Δ *rpn4*Δ, *mub1*Δ *rpn4*Δ, and *rpn4*Δ cells, driven by the native *RPN4* promoter (P*_RPN4_-RPN4*) or a modified *RPN4* promoter with PACE either deleted (P*_RPN4d_-RPN4*) or mutated (P*_RPN4m_-RPN4*), to evaluate the effect of PACE on the expression of *RPN4* and the four efflux pump genes. qPCR analyses showed robust upregulation of all the tested genes in the *ubr2*Δ and *mub1*Δ background only when *RPN4* expression was driven by P*_RPN4_-RPN4* but not P*_RPN4d_-RPN4* or P*_RPN4m_-RPN4* (Fig. 3D). The upregulation was not detected in WT cells where Rpn4 is rapidly degraded. Consistently, deleting or mutating the PACE element dramatically decreased the resistance of *ubr2*Δ and *mub1*Δ cells to fluconazole but had no effect in WT cells (Fig. 3E). Overall, our results prove that the PACE element in its promoter mediates the autoactivation of *RPN4* expression, which further activates the transcription of efflux pump genes to confer fluconazole resistance in *C. auris* (Fig. 3F).

### Rpn4 directly activates *CDR1* transcription via a PACE element

Next, we asked whether Rpn4 regulates *CDR1* and *MDR1* directly or indirectly. As mentioned in the Introduction, overexpression of *CDR1* and *MDR1* is predominantly due to gain-of-function mutations in the zinc cluster transcription factors such as *TAC1* and *MRR1* in *C. albicans* (30–33) and *PDR1* in *C. glabrata* (51). Although no *PDR1* homolog was identified by phylome search in the available genome assemblies for *C. auris*, two *TAC1* and three *MRR1* homologs have been identified and designated as *TAC1a* (*004819*), *TAC1b* (*004820*), *MRR1a* (*004061*), *MRR1b* (*002931*), and *MRR1c* (*004353*) in *C. auris*. Notably, our RNA-Seq data identified a modest increase in *TAC1b*, *MRR1a*, and *MRR1b* expression in *ubr2*Δ and *mub1*Δ cells compared to WT cells (Data S1), although the upregulation of *MRR1a* (log_2_ fold change = 0.85) and *MRR1b* (log_2_ fold change = 0.84) in *mub1*Δ cells did not reach the criteria assigned for DEGs. Thus, we speculated that Rpn4 might increase the expression of *CDR1* and *MDR1* in a *TAC1-* and *MRR1*-dependent way, resulting in fluconazole resistance.

**Fig 4:**
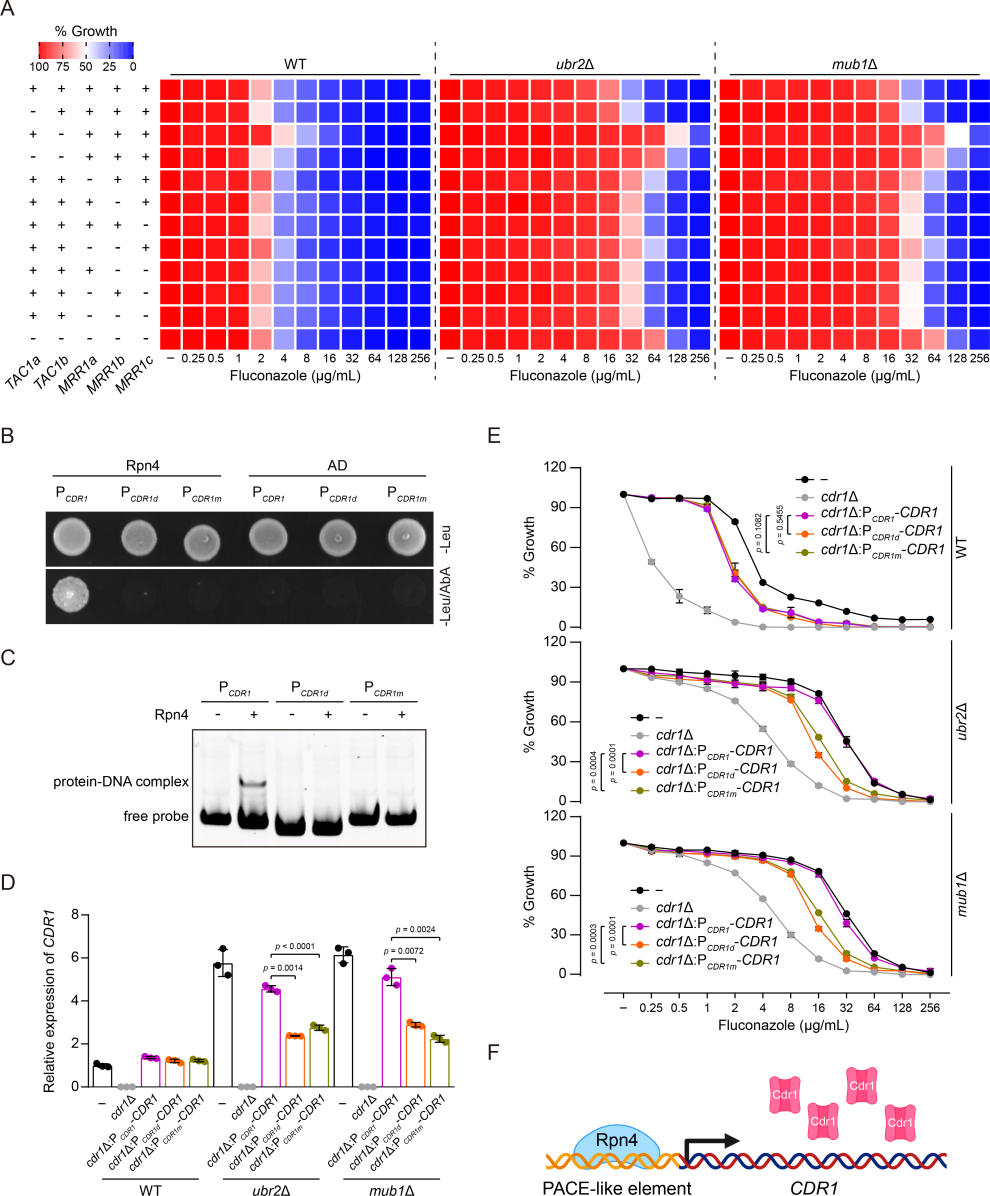
Rpn4 binds to a PACE-like element within the *CDR1* promoter to activate *CDR1* expression. (**A**) Fluconazole susceptibility assays for the indicated strains were performed as described in Fig. 1A. Fluconazole was applied as a twofold dilution series. Growth was measured and normalized to no-drug control. Data are representative of three technical replicates. (**B**) Rpn4 binds to the *CDR1* promoter in the yeast one-hybrid assay. The combinations of pAbAi-P*_CDR1_*, pAbAi-P*_CDR1d_*, or pAbAi-P*_CDR1m_* with the empty prey (AD) vector served as negative controls. (**C**) Gel mobility shift assay was performed by incubating the WT (P*_CDR1_*), PACE element-deleted (P*_CDR1d_*), or -mutated (P*_CDR1m_*) *CDR1* promoter fragments with the purified recombinant Rpn4 produced in *E. coli*. The formation of protein-DNA complex was analyzed using a 6% nondenaturing polyacrylamide gel with SYBR Green staining. (**D**) qPCR analysis of *CDR1* expression in the indicated strains. The transcript level of *CDR1* was normalized to *GPD1* and set to 1 in WT cells. Error bars, SD from the mean of three independent experiments. Significance was measured using two-tailed unpaired Student’s *t*-test. (**E**) Fluconazole susceptibility assays for the indicated strains were performed as described in Fig. 1A. Fluconazole was applied as a twofold dilution series. Growth was measured and normalized to no-drug control. Error bars are the SD from the mean of three technical replicates. Significance was determined using two-way analysis of variance with the Geisser-Greenhouse correction. (**F**) Illustration of the results described in Fig. 4. Rpn4 activates *CDR1* expression by directly binding to a PACE-like element in its promoter, leading to fluconazole resistance in *C. auris*.

To determine whether the *TAC1* and *MRR1* homologs have a role in the high fluconazole resistance caused by Rpn4, we constructed a series of deletion mutants in *ubr2*Δ, *mub1*Δ, and WT cells in which *TAC1a*, *TAC1b*, *MRR1a*, *MRR1b*, and *MRR1c* were deleted individually or in combination. If these transcription factors bridge Rpn4’s activity in upregulating efflux pump genes, their deletion should decrease or eliminate the fluconazole resistance of *ubr2*Δ and *mub1*Δ cells. We found that deleting *TAC1a* or any *MRR1* homolog either had no or only a slight effect on fluconazole susceptibility compared with their respective parental strains (Fig. 4A). Interestingly, the loss of *TAC1b* caused a significant increase of fluconazole resistance (Fig. 4A), indicating that *TAC1b* may act as a transcriptional repressor of *CDR1*. This is consistent with previous reports that implicate the differential roles of Tac1b in *C. auris* azole resistance between clades. In clade III and clade IV isolates, deletion of *TAC1b* caused a decrease in fluconazole and voriconazole resistance without conspicuously dysregulating the expression of *CDR1* (35). In contrast, many fluconazole-resistant isolates from clade I acquired gain-of-function mutations in *TAC1b* with a significant upregulation of *CDR1* expression (34). The observation that fluconazole susceptibility was not significantly affected by the absence of *TAC1a*,*b* and *MRR1a*,*b*,*c* suggested that Rpn4 might directly target *CDR1* and *MDR1*.

Inspection of *CDR1* and *MDR1* promoter regions identified a PACE-like element (5´-GGCGGCAAA-3´) located at n.t. −162 to −154 upstream of the START codon of *CDR1*, which differs from the consensus PACE element (5´-GGTGGCAAA-3´) by one nucleotide. However, we did not detect any potential Rpn4 binding site in the *MDR1* promoter. We, therefore, investigated whether Rpn4 directly binds to the *CDR1* promoter via this PACE-like element. First, yeast one-hybrid (Y1H) analysis demonstrated Rpn4 interaction with the *CDR1* promoter (P*_CDR1_*) (Fig. 4B). This interaction was abolished by either deleting the entire PACE motif (P*_CDR1d_*) or mutating it to 5´-AAGAATAAA-3´ (P*_CDR1m_*) (Fig. 4B), indicating PACE-dependent interaction. Consistently, incubation with purified recombinant Rpn4 from *E. coli* retarded the mobility of the *CDR1* promoter fragment carrying the PACE element (P*_CDR1_*) in the electrophoretic mobility shift assay (Fig. 4C). In contrast, mobility retardation was not observed when PACE was deleted (P*_CDR1d_*) or mutated (P*_CDR1m_*) (Fig. 4C). Together, these results demonstrate that the PACE element is required for recruiting Rpn4 to the *CDR1* promoter.

Next, we asked whether Rpn4 drives *CDR1* expression by binding to the PACE motif in the *CDR1* promoter. We complemented *ubr2*Δ *cdr1*Δ, *mub1*Δ *cdr1*Δ, and *cdr1*Δ mutants with the full-length *CDR1* controlled by the WT *CDR1* promoter (P*_CDR1_-CDR1*) or a modified *CDR1* promoter with PACE either deleted (P*_CDR1d_-CDR1*) or mutated (P*_CDR1m_-CDR1*). Deletion or mutation of PACE had no noticeable impact on the transcript level of *CDR1* in the WT background (Fig. 4D). In contrast, the two mutated *CDR1* promoters dramatically reduced the induction of *CDR1* expression by Rpn4 compared to the WT promoter in the *ubr2*Δ and *mub1*Δ background (Fig. 4D). Congruously, the PACE-deleted or -mutated *CDR1* promoters rendered *ubr2*Δ and *mub1*Δ cells but not WT cells more susceptible to fluconazole (Fig. 4E). Thus, these results show that the direct binding of Rpn4 to the PACE element within the *CDR1* promoter is essential for activating *CDR1* expression to confer fluconazole resistance in *C. auris* (Fig. 4F).

### Identification of an A316T mutation in *UBR2* in *C. auris* clinical isolates that confers fluconazole resistance via the Rpn4-efflux pump axis

**Fig 5:**
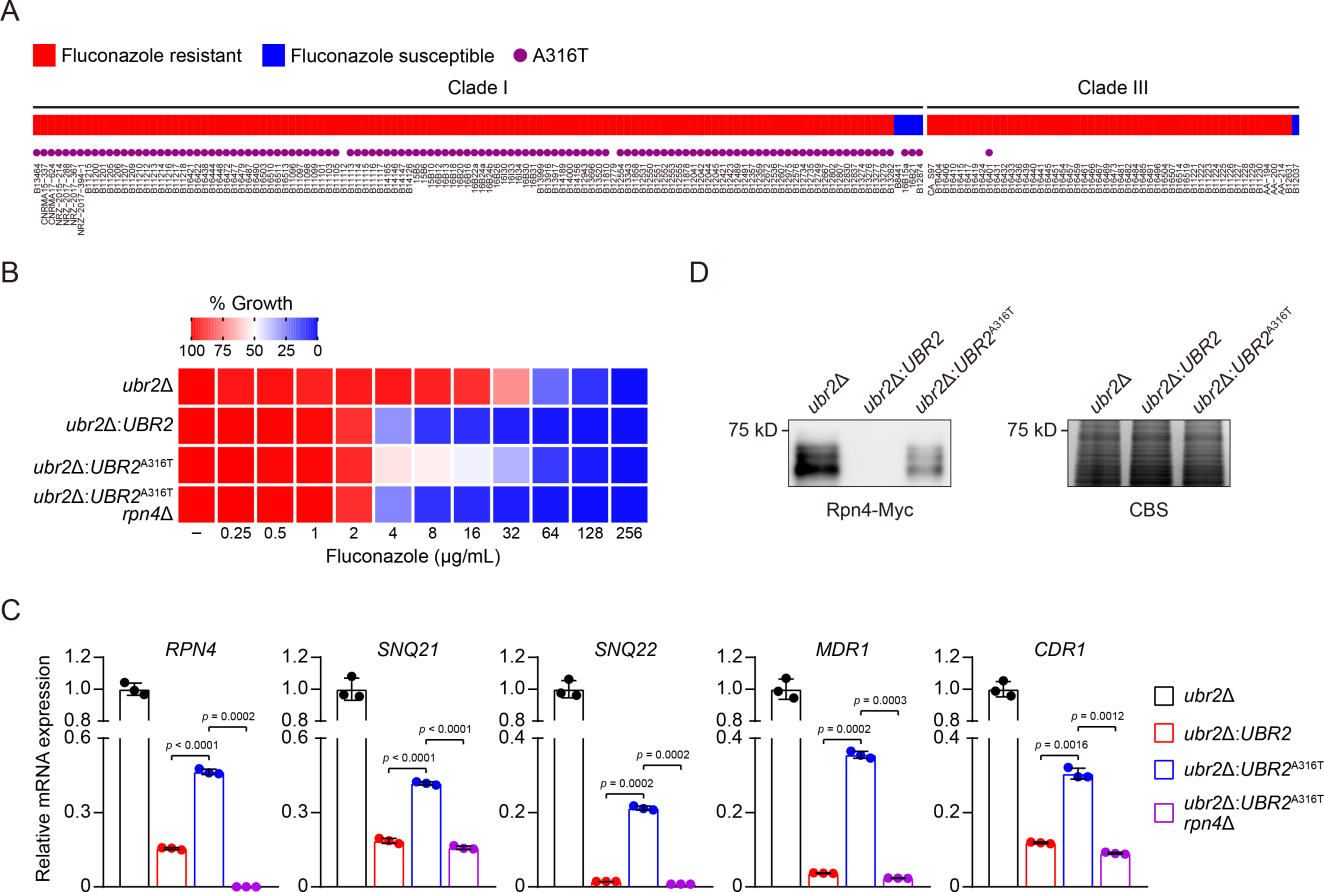
The *UBR2*^A316T^ mutation reduces fluconazole susceptibility via Rpn4-mediated upregulation of efflux pump gene expression. (**A**) *C. auris* clinical isolates from clade I and III with an A316T mutation in *UBR2*. The resistant and susceptible isolates are indicated as red and blue boxes, respectively. The purple dots indicate isolates harboring the *UBR2*^A316T^ mutation. The strain name of each isolate is listed at the bottom of this panel. (**B**) Fluconazole susceptibility assays for the indicated strains were performed as described in Fig. 1A. Fluconazole was applied as a twofold dilution series. Growth was measured and normalized to no-drug control. Data are representative of three technical replicates. (**C**) qPCR analysis of *RPN4*, *SNQ21*, *SNQ22*, *MDR1*, and *CDR1* expressions in the indicated strains. Transcript levels were normalized to *GPD1*. The transcript level of each gene in *ubr2*Δ cells was set to 1. Error bars, SD from the mean of three independent experiments. Significance was determined using two-tailed unpaired Student’s *t*-test. (**D**) The protein level of endogenously expressed Rpn4-Myc in *ubr2*Δ, *ubr2*Δ:*UBR2*, and *ubr2*Δ:*UBR2*^A316T^ cells. Coomassie Blue staining (CBS) of a replicate gel served as loading control.

Inspired by the contribution of the Ubr2/Mub1-Rpn4-efflux pump signaling pathway to *C. auris* fluconazole resistance, we next attempted to assess its clinical significance by analyzing a whole-genome sequencing data collection, which includes 304 *C*. *auris* clinical isolates representing each of the four major genetic clades (4). We identified a total of 2,957 fluconazole resistance-associated genotypic variations involving several well-defined changes in *ERG11* and *TAC1b* (Data S3). Interestingly, we also found 25, 23, and 3 mutations in *UBR2*, *MUB1*, and *RPN4*, respectively (Fig. S4A). Notably, a *UBR2* A316T mutation was found among 98% (119/122) of the most highly resistant clade I isolates and one clade III isolate (Fig. 5A). Protein complex prediction using AlphaFold2-Multimer (52) revealed that the A316T mutation leads to a shift of the original α-helix due to the β-carbon atom of threonine (Fig. S4B). We reasoned that this structural change might alter the interaction between Rpn4 and the Mub1/Ubr2 ubiquitin-ligase and affect Rpn4 degradation, therefore increasing the cellular level of Rpn4, efflux pump gene expression, and fluconazole resistance.

To experimentally test this hypothesis, we introduced the *UBR2* allele containing the A316T substitution into *ubr2*Δ cells (*ubr2*Δ:*UBR2*^A316T^). While more sensitive to fluconazole than *ubr2*Δ cells, this strain showed a modest increase in fluconazole resistance compared to *ubr2*Δ:*UBR2* cells (Fig. 5B). This effect depended on Rpn4 since deleting *RPN4* abolished the increased fluconazole resistance in *ubr2*Δ:*UBR2*^A316T^ cells (Fig. 5B). The results demonstrate a role for Rpn4 in fluconazole resistance caused by the *UBR2*^A316T^ mutation. Furthermore, we observed an *RPN4*-dependent increase in the expression of four efflux pump genes (*CDR1*, *SNQ21*, *SNQ22*, and *MDR1*), as well as that of *RPN4* in *ubr2*Δ:*UBR2*^A316T^ cells compared to *ubr2*Δ:*UBR2* cells, which was abolished by deleting *RPN4* (Fig. 5C). However, the magnitude of the increase was lower in *ubr2*Δ:*UBR2*^A316T^ cells compared to *ubr2*Δ cells (Fig. 5C), consistent with our observation that *ubr2*Δ cells displayed higher fluconazole resistance than *ubr2*Δ:*UBR2*^A316T^ cells. This difference is reasonable because the *UBR2*^A316T^ mutation may merely weaken the activity of the Ubr2/Mub1 ubiquitin-ligase to degrade Rpn4, in contrast to the complete elimination of this process in *ubr2*Δ cells. Indeed, while Rpn4 is undetectable by immunoblotting in *ubr2*Δ:*UBR2* cells, it can be detected in *ubr2*Δ:*UBR2*^A316T^ cells, although its abundance is lower than that in *ubr2*Δ cells (Fig. 5D). Together, our results strongly suggest that the *UBR2*^A316T^ substitution represents a clinically relevant mutation that results in diminished fluconazole susceptibility through Rpn4-mediated upregulation of *CDR1* and other drug transporters. These findings underscore the significance of this Rpn4-efflux pump axis in the fluconazole resistance of *C. auris*.

## DISCUSSION

**Fig 6:**
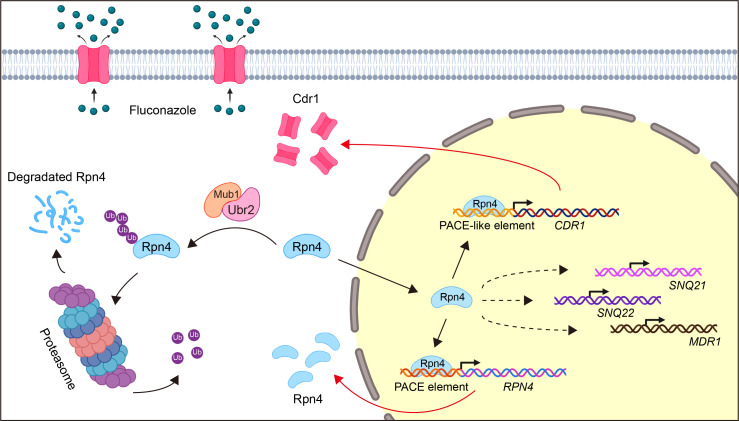
Model depicting a positive *RPN4* feedback loop in regulating fluconazole resistance in *C. auris*. The Mub1/Ubr2 ubiquitin-ligase complex degrades Rpn4 and represses its function. Inhibition of either Mub1 or Ubr2 leads to an accumulation of Rpn4 through a positive feedback loop: Rpn4 turns on its own transcription by binding to the PACE element in its promoter. *RPN4* autoregulation is essential for subsequent transcriptional activating of efflux genes to confer fluconazole resistance, especially *CDR1*. Induction of *CDR1* depends on the direct binding of Rpn4 to the PACE element within the *CDR1* promoter.

Over the past decade, *C. auris* has emerged as one of the most dangerous human fungal pathogens. It is one of the only four pathogens classified in the critical priority group on the fungal priority pathogens list recently released by the World Health Organization (53). The primary reason is its intrinsic resistance to fluconazole and its propensity to develop resistance to other antifungals rapidly as well (7). Several studies have associated fluconazole resistance in many *C. auris* clinical isolates with the overexpression of the ABC efflux transporter gene *CDR1* and mutations in the *ERG11* gene that encodes the drug target sterol 14α-demethylase (4, 7, 20–29). Studies in other human fungal pathogens have identified several master regulators responsible for the upregulation of *CDR1*, such as the transcription factors Tac1 of *C. albicans* (30, 31) and Pdr1 of *C. glabrata* (51). Genomic analyses have revealed the presence of two *TAC1* homologs, *TAC1a* and *TAC1b*, in the *C. auris* genome, and some mutations in *TAC1b* have been shown to contribute to fluconazole resistance (22, 34, 54). However, there is currently no experimental evidence to substantiate that *TAC1b* mutations cause fluconazole resistance by promoting *CDR1* overexpression. Conversely, a study indicated that the loss of *TAC1b* did not affect *CDR1* transcript levels (35), suggesting the existence of an unknown transcription factor(s) that regulates *CDR1* expression. In this study, we demonstrate that the transcription factor Rpn4 activates its expression through a positive autoregulatory loop, which further promotes the expression of a set of efflux pump genes, particularly *CDR1*, conferring fluconazole resistance in *C. auris* (Fig. 6). The regulatory target(s) of Tac1b remains an open question for future investigations.

Currently, studies of *C. auris* rely heavily on inferences drawn from knowledge and data obtained in the studies of the model human fungal pathogen *C. albicans*. Given the distant phylogenetic relationship and significant variations in clinically relevant traits between them, this approach inevitably misses out on genes that perform specific functions and determine unique traits in *C. auris*. For example, the antifungal resistance mechanisms learned from *C. albicans* often fail to explain the resistance phenotype in many *C. auris* isolates, suggesting considerable divergence in resistance mechanisms between these two *Candida* species and emphasizing the need for detailed mechanistic characterization of *C. auris* antifungal resistance. Several recently developed unbiased genome-wide genetic screen tools have provided powerful solutions to this problem and identified new genes regulating pathogenic properties in *C. auris* (36, 55). We constructed the *piggyBac* transposon-mediated mutagenesis system in *C. auris* and identified a novel global stress regulator *DINOR* that regulates drug resistance and stress response (36). Also, the Agrobacterium-mediated transformation system and CRISPR (clustered regularly interspaced short palindromic repeats)-Cas9 system have been developed and applied successfully to identify genes that control *C. auris* morphogenesis (55).

This study has revealed a signaling pathway that plays an important role in fluconazole resistance in *C. auris*. We found that mutating the *MUB1* gene caused high resistance to fluconazole (Fig. 1A). In *S. cerevisiae*, Mub1 forms a complex with the E3 ubiquitin-ligase Ubr2 required for the ubiquitination and degradation of the transcription factor Rpn4 (37, 39). Our results demonstrate that this cellular process is conserved in *C. auris*. We observed a marked increase in the Rpn4 protein level in both *ubr2*Δ and *mub1*Δ mutants (Fig. 1B, C, and F). Deletion of *RPN4* abolished fluconazole resistance in these two mutants (Fig. 1H), indicating an essential role of Rpn4 in regulating this drug resistance phenotype. Although *RPN4* has been implicated in fluconazole resistance in other fungal species, in no case was Rpn4 shown to promote the expression of efflux pumps directly. For example, *RPN4* was found to be directly regulated by the multidrug resistance regulators Pdr1 and Pdr3 in *S. cerevisiae* (50). However, based on the well-known role of Rpn4 in regulating proteosome genes, the authors speculated that Rpn4 contributes to drug resistance by promoting the degradation of misfolded proteins caused by toxic compounds. Two other studies only demonstrated increased sensitivity to fluconazole in *rpn4*Δ mutants without any mechanistic explanation (56, 57). Similarly, increased fluconazole sensitivity was observed in a *rpn4*Δ mutant in *C. albicans* during the phenotypic profiling of a transcription factor mutant library (58). In *C. glabrata*, *RPN4* overexpression and point mutation have been detected in several fluconazole-resistant isolates (59). Recently, Pais et al. found that Rpn4 regulates fluconazole resistance by directly activating the expression of several genes of the ergosterol biosynthetic pathway and excluded a role for Rpn4 in activating the expression of drug transporters (42). In this study of *C. auris*, we obtained illuminating clues about Rpn4’s mechanism of action in fluconazole resistance by performing a global transcriptomic comparison of *mub1*Δ, *ubr2*Δ, and WT strains, detecting the upregulation of four predicted efflux pump genes (23), *SNQ21*, *SNQ22*, *MDR1* and *CDR1* (Fig. 2A and E), previously reported to be involved in drug resistance in other fungal pathogens (60). Subsequent gene deletion experiments revealed that Cdr1 plays a dominant role in fluconazole resistance compared with other efflux transporters (Fig. 2G), consistent with previous reports that *CDR1* is a primary contributor to fluconazole resistance in *C. auris* (26, 27). To our knowledge, Rpn4 is the first reported *C. auris* transcription factor that directly regulates efflux gene transcription. This is different from the regulatory pathway previously described for *S. cerevisiae* (50, 61). It remains unknown whether the Ubr2/Mub1-Rpn4-Cdr1 pathway also determines fluconazole resistance in other fungal pathogens. Previous data and this study’s findings indicate that different fungal pathogens have evolved distinct mechanisms involving Rpn4 to regulate resistance to fluconazole. The reconfiguration of signaling pathways that connect a transcription factor to various downstream effectors, enabling resistance to antifungal drugs, underscores the adaptability of fungal pathogens in facing and enduring external challenges.

In eukaryotes, positive transcriptional autoregulation has been observed for many transcription factors, playing essential roles in numerous biological processes, such as cell growth, differentiation, and development (62). Our findings demonstrate that Rpn4 forms a positive autoregulatory loop by directly binding to a PACE element in its own promoter, likely ensuring rapid Rpn4 synthesis and maintenance of its cellular level to activate and sustain the expression of efflux genes (Fig. 3A through D). As expected, fluconazole resistance depends on *RPN4* positive autoregulation (Fig. 3E). Consistently, similar rapid transcriptional autoregulation of Tac1 and Pdr1 in mediating fluconazole resistance has been observed in *C. albicans* (63) and *C. glabrata* (64), respectively. Also, we found several lines of evidence indicating that Rpn4 activates *CDR1* transcription by binding to a PACE motif in the *CDR1* promoter. First, *RPN4* is required for *CDR1* upregulation in *ubr2*Δ and *mub1*Δ cells (Fig. 2E). Second, Rpn4 expressed in yeast or isolated from bacteria can bind to the PACE motif (Fig. 4B and C). Third, deleting or mutating PACE in the *CDR1* promoter abolishes the Rpn4-dependent *CDR1* overexpression (Fig. 4D). Fourth, deletion or mutation of the PACE sequence reduces *RPN4*-dependent fluconazole resistance (Fig. 4E). Although we have not identified putative Rpn4-binding motifs in the promoter region of other efflux genes, such as *MDR1*, *SNQ21*, and *SNQ22*, we cannot exclude the possibility that *C. auris* Rpn4 can also recognize other motifs to turn on transcription. Further experiments are needed to map the Rpn4-binding sites in these promoters and determine their functions. Together, our findings strongly support the notion that Rpn4 is an important determinant of fluconazole resistance by directly activating the expression of the ABC transporter gene *CDR1* and possibly other transporter genes in *C. auris*. This new knowledge, together with *ERG11* mutations, *TAC1b* gain-of-function mutations, and *MRR1* mutations, may explain the multifactorial and complex mechanisms of fluconazole resistance in *C. auri*s.

So far, the few well-established mechanisms of antifungal drug resistance have served us well in pinpointing mutations responsible for drug resistance in many clinical isolates (20–22, 24, 34). However, they have also narrowly directed our attention to a few genes, frequently leading to repetitive reports of the same resistance mechanisms. Whole genome-sequencing analyses of resistant isolates always reveal numerous SNPs and other mutations, often failing to go far if mutations are not found in the usual suspects. In addition, the new drug resistance regulatory mechanisms recently revealed by unbiased genome-wide genetic screens strongly suggest that the few known resistance mechanisms may only represent the tip of an iceberg (36, 65, 66). Thus, findings made in this study and other similar studies provide helpful guidance for future targeted searches for mutations responsible for drug resistance in clinical isolates, because *C. auris*, like *C. glabrata*, has a haploid genome, a single mutation has a high probability of producing a significant phenotype. By analyzing the genome sequences of 304 *C*. *auris* clinical isolates from 19 countries on six continents (4), we identified a *UBR2* mutation that encodes the A316T substitution. We have shown that this mutation reduces fluconazole susceptibility by activating the Rpn4-efflux pump axis (Fig. 5). Our findings demonstrate that mutations resulting in Rpn4 stabilization may serve as potent genetic determinants of clinical fluconazole resistance in *C. auris*. Continued identification and characterization of additional mutations that contribute to Rpn4-associated fluconazole resistance may help elucidate the substantial resistance that cannot be explained by known resistance mechanisms in *C. auris*. In future studies, the increasing use of unbiased screening approaches in combination with genotypic analysis of clinical isolates will generate a complete picture of drug resistance mechanisms in fungal pathogens, which is essential for developing effective antifungal strategies to conquer multidrug-resistant fungal pathogens.

## MATERIALS AND METHODS

### Strains, media, and growth conditions

*C. auris* strain CBS10913 was used as the WT strain for all experiments unless otherwise noted. All *C. auris* strains derived from CBS10913 and used in this study are listed in Table S1. To validate a mutant phenotype and exclude any unlinked mutational effects, we constructed two independent strains for all mutants presented in this study. *C. auris* was routinely grown in YPD medium (1% yeast extract, 2% tryptone, and 2% glucose) or in Glucose Minimal Medium (GMM) (6.79 g/L yeast nitrogen base without amino acids and 2% glucose) at 30°C. For solid media, 2% agar was added. Transformants were selected on YPD plates containing 250 µg/mL nourseothricin (Jena Bioscience, AB-102) or 1 mg/mL hygromycin B (Sigma-Aldrich, H7772) or GMM agar plates.

### Genetic screens using *PB*-mediated mutagenesis system

To screen for fluconazole-resistant mutants, transposition-mediated mutagenesis was performed using the CauW08 strain grown on YPD plate containing 50 µg/mL Dox for 2 days at 30°C. Then, the cells were replica transferred to GMM plates and incubated at 30°C for 2 days to generate the mutant library. Subsequently, mutant cells were plated onto the YPD medium containing 60 µg/mL fluconazole and incubated at 30°C until visible resistant colonies appeared with minimal background growth.

### Gene disruption

Target gene deletion was conducted using homologous recombination based on the fusion PCR method together with the *SAT1* flipping strategy. The *LEU2* nutritional marker was PCR amplified from *C. auris* genomic DNA. The *SAT1* selectable marker was PCR amplified from the pNIM1-CaPBase plasmid (66). The *HYG* selectable marker was PCR amplified from a plasmid generated by Dr. Huang Guanghua’s lab. The 3´ end of the upstream PCR product and the 5´ end of the downstream PCR product include short homologies to the selectable markers for fusion PCR to generate the deletion cassette. The deletion plasmids harboring the *SAT1* flipper cassette were constructed by inserting the upstream homologous fragments between the *Sph*I/*BamH*I restriction sites and the downstream homologous fragments between the *Not*I/*Sac*II restriction sites, respectively. Phusion Hot Start II DNA Polymerase (Thermo Fisher Scientific, F549L) was used in all PCR reactions. PCR products were purified using the E.Z.N.A. Gel Extraction Kit (Omega Bio-tek, D2500-02) after agarose gel electrophoresis. Primer sequences for gene deletion are listed in Table S2.

### Strain construction

To disrupt *MUB1*, the upstream homology arm was amplified using the primer pair MUB1uF-MUB1LuR for the *LEU2* marker, MUB1uF-MUB1SuR for the *SAT1* marker, or MUB1uF-MUB1HuR for the *HYG* marker. The downstream homology arm was amplified with the primer pair MUB1LdF-MUB1dR for the *LEU2* marker, MUB1SdF-MUB1dR for the *SAT1* marker, or MUB1HdF-MUB1dR for the *HYG* marker. The interior primer of each set contains a 15 bp homologous region to the selectable marker which was amplified with the primer pair MUB1LF-MUB1LR for the *LEU2* marker, MUB1SF-MUB1SR for the *SAT1* marker, or MUB1HF-MUB1HR for the *HYG* marker. Finally, the linear deletion cassette with homology arms flanking the selectable marker was amplified using fusion PCR with the primer pair MUB1uF-MUB1dR. The integration of the deletion cassette was verified using primer pair MUB1F-LEU2R, MUB1F-SAT1R, or MUB1F-HYGR. The disruption of the WT allele was verified using the primer pair MUB1F- MUB1kR.

To disrupt *UBR2*, the upstream homology arm was amplified using the primer pair MUB1uF-MUB1LuR for the *LEU2* marker, UBR2uF-UBR2SuR for the *SAT1* marker, or UBR2uF-UBR2HuR for the *HYG* marker. The downstream homology arm was amplified with the primer pair UBR2LdF-UBR2dR for the *LEU2* marker, UBR2SdF-UBR2dR for the *SAT1* marker, or UBR2HdF-UBR2dR for the *HYG* marker. The interior primer of each set contains a 15 bp homologous region to the selectable marker which was amplified with the primer pair UBR2LF-UBR2LR for the *LEU2* marker, UBR2SF-UBR2SR for the *SAT1* marker, or UBR2HF-UBR2HR for the *HYG* marker. Finally, the linear deletion cassette comprising homology arms flanking the selectable marker was amplified using fusion PCR with the primer pair UBR2uF-UBR2dR. The integration of the deletion cassette was verified using primer pair UBR2F-LEU2R, UBR2F-SAT1R, or UBR2F-HYGR. The disruption of the WT allele was verified using the primer pair UBR2F-UBR2kR.

To disrupt *RPN4*, the upstream homology arm was amplified using the primer pair RPN4uF-RPN4SuR for the *SAT1* marker or RPN4uF-RPN4HuR for the *HYG* marker. The downstream homology arm was amplified with the primer pair RPN4SdF-RPN4dR for the *SAT1* marker or RPN4HdF-RPN4dR for the *HYG* marker. The interior primer of each set contains a 15 bp homologous region to the selectable marker which was amplified with the primer pair RPN4SF-RPN4SR for the *SAT1* marker or RPN4HF-RPN4HR for the *HYG* marker. Finally, the linear deletion cassette containing homology arms flanking the selectable marker was amplified using fusion PCR with the primer pair RPN4uF-RPN4dR. The integration of the deletion cassette was verified using the primer pair RPN4F-SAT1R or RPN4F-HYGR. The disruption of the WT allele was verified using the primer pair RPN4F-RPN4kR.

To disrupt *MDR1*, the upstream homology arm was amplified using the primer pair MDR1uF-MDR1SuR for the *SAT1* marker or MDR1uF-MDR1HuR for the *HYG* marker. The downstream homology arm was amplified with the primer pair MDR1SdF-MDR1dR for the *SAT1* marker or MDR1HdF-MDR1dR for the *HYG* marker. The interior primer of each set contains a 15 bp homologous region to the selectable marker, which was amplified with the primer pair MDR1SF-MDR1SR for the *SAT1* marker or MDR1HF-MDR1HR for the *HYG* marker. Finally, the linear deletion cassette incorporating homology arms flanking the selectable marker was amplified using fusion PCR with the primer pair MDR1uF-MDR1dR. The integration of the deletion cassette was verified using primer pair MDR1F-SAT1R or MDR1F-HYGR. The disruption of the WT allele was verified using the primer pair MDR1F-MDR1kR.

To disrupt *CDR1*, the upstream homology arm was amplified using the primer pair CDR1uF-CDR1LuR for the *LEU2* marker or CDR1uF-CDR1SuR for the *SAT1* marker. The downstream homology arm was amplified with the primer pair CDR1LdF-CDR1dR for the *LEU2* marker or CDR1SdF-CDR1dR for the *SAT1* marker. The interior primer of each set contains a 15 bp homologous region to the selectable marker which was amplified with the primer pair CDR1LF-CDR1LR for the *LEU2* marker or CDR1SF-CDR1SR for the *SAT1* marker. Finally, the linear deletion cassette with homology arms flanking the selectable marker was amplified using fusion PCR with the primer pair CDR1uF-CDR1dR. The integration of the deletion cassette was verified using primer pair CDR1F-LEU2R or CDR1F-SAT1R. The disruption of the WT allele was verified using the primer pair CDR1F-CDR1kR.

To disrupt *SNQ21*, the upstream homology arm was amplified using the primer pair SNQ21uF-SNQ21LuR. The downstream homology arm was amplified with the primer pair SNQ21LdF-SNQ21dR. The interior primer of each set contains a 15 bp homologous region to the *LEU2* marker which was amplified with the primer pair SNQ21LF-SNQ21LR. Finally, the linear deletion cassette incorporating homology arms flanking the *LEU2* marker was amplified using fusion PCR with the primer pair SNQ21uF-SNQ21dR. The integration of the deletion cassette was verified using the primer pair SNQ21F-LEU2R. Otherwise, the upstream and downstream homology arm was amplified with primer pairs SNQ21SFuF-SNQ21SFuR and SNQ21SFdF-SNQ21SFdR, which were then inserted into a plasmid containing the *SAT1* flipper cassette using the *Sph*I/*BamH*I and *Not*I/*Sac*II restriction sites, respectively. Finally, the linear deletion cassette incorporating homology arms flanking the *SAT1* flipper cassette was generated by digesting the plasmid with *Sph*I/*Sac*II. The disruption of the WT allele was verified using the primer pair SNQ21F-SNQ21kR.

To disrupt *SNQ22*, the upstream homology arm was amplified using the primer pair SNQ22uF-SNQ22LuR. The downstream homology arm was amplified with the primer pair SNQ22LdF-SNQ22dR. The interior primer of each set contains a 15 bp homologous region to the *LEU2* marker which was amplified with the primer pair SNQ22LF-SNQ22LR. Finally, the linear deletion cassette incorporating homology arms flanking the *LEU2* marker was amplified using fusion PCR with the primer pair SNQ22uF-SNQ22dR. The integration of the deletion cassette was verified using the primer pair SNQ22F-LEU2R. Otherwise, the upstream and downstream homology arms were amplified with primer pairs SNQ22SFuF-SNQ22SFuR and SNQ22SFdF-SNQ22SFdR, which were then inserted into a plasmid containing the *SAT1* flipper cassette using the *Sph*I/*BamH*I and *Not*I/*Sac*II restriction sites, respectively. Finally, the linear deletion cassette incorporating homology arms flanking the *SAT1* flipper cassette was generated by digesting the plasmid with *Sph*I/*Sac*II. The disruption of the WT allele was verified using the primer pair SNQ22F-SNQ22kR.

To disrupt *TAC1a*, the upstream and downstream homology arms were amplified with primer pairs TAC1aSFuF-TAC1aSFuR and TAC1aSFdF-TAC1aSFdR, which were then inserted into a plasmid containing the *SAT1* flipper cassette at the *Sph*I/*BamH*I and *Not*I/*Sac*II restriction sites, respectively. Finally, the linear deletion cassette incorporating homology arms flanking the *SAT1* flipper cassette was generated by digesting the plasmid with *Sph*I/*Sac*II. The disruption of the WT allele was verified using the primer pair TAC1aF-TAC1akR.

To disrupt *TAC1b*, the upstream and downstream homology arms were amplified with primer pairs TAC1bSFuF-TAC1bSFuR and TAC1bSFdF-TAC1bSFdR, which were then inserted into a plasmid containing the *SAT1* flipper cassette at the *Sph*I/*BamH*I and *Not*I/*Sac*II restriction sites, respectively. Finally, the linear deletion cassette incorporating homology arms flanking the *SAT1* flipper cassette was generated by digesting the plasmid with *Sph*I/*Sac*II. The disruption of the WT allele was verified using the primer pair TAC1bF-TAC1bkR.

To disrupt *MRR1a*, the upstream and downstream homology arms were amplified with primer pairs MRR1aSFuF-MRR1aSFuR and MRR1aSFdF-MRR1aSFdR, which were then inserted into a plasmid containing the *SAT1* flipper cassette at the *Sph*I/*BamH*I and *Not*I/*Sac*II restriction sites, respectively. Finally, the linear deletion cassette incorporating homology arms flanking the *SAT1* flipper cassette was generated by digesting the plasmid with *Sph*I/*Sac*II. The disruption of the WT allele was verified using the primer pair MRR1aF-MRR1akR.

To disrupt *MRR1b*, the upstream and downstream homology arms were amplified with primer pairs MRR1bSFuF-MRR1bSFuR and MRR1bSFdF-MRR1bSFdR, which were then inserted into a plasmid containing the *SAT1* flipper cassette at the *Sph*I/*BamH*I and *Not*I/*Sac*II restriction sites, respectively. Finally, the linear deletion cassette incorporating homology arms flanking the *SAT1* flipper cassette was generated by digesting the plasmid with *Sph*I/*Sac*II. The disruption of the WT allele was verified using the primer pair MRR1bF-MRR1bkR.

To disrupt *MRR1c*, the upstream and downstream homology arms were amplified with primer pairs MRR1cSFuF-MRR1cSFuR and MRR1cSFdF-MRR1cSFdR, which were then inserted into a plasmid containing the *SAT1* flipper cassette at the *Sph*I/*BamH*I and *Not*I/*Sac*II restriction sites, respectively. The linear deletion cassette incorporating homology arms flanking the *SAT1* flipper cassette was generated by digesting the plasmid with *Sph*I/*Sac*II. The disruption of the WT allele was verified using the primer pair MRR1cF-MRR1ckR.

The complemented strain *mub1*Δ:*MUB1* was constructed by introducing the WT *MUB1* copy into *mub1*Δ cells at the endogenous *MUB1* locus upstream of the *LEU2* disruption cassette. The complemented strain *ubr2*Δ:*UBR2* and *ubr2*Δ:*UBR2*^A316T^ was constructed similarly. The complementation of *MUB1* and *UBR2* was verified using primer pairs MUB1F-MUB1kR and UBR2F-UBR2kR, respectively.

The strains expressing proteins fused with different N-terminal or C-terminal tags (Myc, GFP, mCherry) under endogenous promoters were constructed by plasmid-based tagging as previously described (67).

The reporter strains used in Fig. 3A were constructed by integrating the *RPN4* promoter-controlled orange fluorescent protein (tdTomato) cassette at the *RPN4* locus of *ubr2*Δ, *mub1*Δ, *rpn4*Δ, *ubr2*Δ *rpn4*Δ, *mub1*Δ *rpn4*Δ, and WT cells using the *HYG* marker.

The complemented strains used in Fig. 3D and E were constructed by introducing full-length *RPN4* driven by the native *RPN4* promoter (P*_RPN4_-RPN4*) or a modified *RPN4* promoter with PACE either deleted (P*_RPN4d_-RPN4*) or mutated (P*_RPN4m_-RPN4*) into *ubr2*Δ *rpn4*Δ, *mub1*Δ *rpn4*Δ, and *rpn4*Δ cells at the *ENO1* locus using the *HYG* marker. The integration of the complementation cassette was verified using the primer pair PENO1F-PJETR.

The complemented strains used in Fig. 4D and E were constructed by introducing full-length *CDR1* controlled by the WT *CDR1* promoter (P*_CDR1_-CDR1*) or a modified *CDR1* promoter with PACE either deleted (P*_CDR1d_-CDR1*) or mutated (P*_CDR1m_-CDR1*) into *ubr2*Δ *cdr1*Δ, *mub1*Δ *cdr1*Δ, and *cdr1*Δ cells at the *ENO1* locus using the *HYG* marker. The integration of the complementation cassette was verified using the primer pair PENO1F-PJETR.

### *C. auris* transformation

*C. auris* transformation was performed using the lithium acetate/heat-shock method. Overnight cultures were centrifuged, washed once each with sterile water, and 1× TE + LiAc buffer (10 mM Tris, 1 mM EDTA, 100 mM lithium acetate), then resuspended in 1× TE + LiAc buffer. Transformation was set up containing thermally denatured salmon sperm DNA (Sigma-Aldrich, D9156), the competent cells, 50% PEG4000 (Sigma-Aldrich, P4338), and the digested plasmid or the amplified gene deletion cassette. The transformation mixture was incubated at 30°C for 6–8 h and mixed by vortexing every 1–2 h. After a 20-min heat-shock at 42°C and 5-min cooling on ice, cells were pelleted, resuspended in YPD medium, and incubated with shaking at 30°C overnight. Cells were then washed twice with sterile water and plated on YPD plates containing 250 µg/mL nourseothricin or 1 mg/mL hygromycin B or GMM plates and incubated at 30°C until transformants appeared.

### Drug susceptibility assay

Drug susceptibility of the indicated strains was assessed by dose-response assays in 96-well plates (Corning, 3799). Approximately 400–500 cells were inoculated into 200 µL of YPD containing twofold serial dilutions of fluconazole (Sigma-Aldrich, F8929). Plates were incubated without shaking at 30°C for 72 h, and then the OD_600_ of each well was measured using a microplate reader (TECAN). The relative growth ratios were calculated by normalizing OD_600_ values against the no-drug controls and were plotted as a heat map using GraphPad Prism except for those in Fig. 4E.

### Protein extraction, IP, and WB analysis

Cells were lysed by beating with ~0.5 mm-diameter glass beads (Sigma-Aldrich, G8772) in the lysis buffer (50 mM Tris pH 7.5, 1 mM EDTA, 1% Triton X-100, 150 mM NaCl) containing 1× complete protease inhibitor cocktail (Roche, 11697498001) using a FastPrep instrument (speed = 4 m/s, time = 5 × 50 s). The cell lysate was cleared by centrifugation. Protein concentration was determined using the Pierce BCA Protein Assay Kit (Thermo Fisher Scientific, 23227).

IP was performed by incubating the supernatant with the Myc-Trap beads (Chromotek, ytma-200) at 4°C overnight. A magnetic stand was used to separate beads from the supernatant. After three washes of the IP products with the lysis buffer, proteins bound to beads were eluted by boiling in 2× Laemmli sample buffer (Bio-Rad, 1610737).

Protein samples were separated by a 10% SDS-PAGE gel and immunoblotted with antibodies. The antibodies used for WB were as follows: anti-Myc mouse monoclonal antibody (Santa Cruz Biotechnology, sc-40), anti-GFP mouse monoclonal antibody (Roche, 11814460001), and horseradish peroxidase (HRP)-conjugated anti-mouse IgG antibody (Santa Cruz Biotechnology, sc-2031). Protein bands were visualized using the Pierce ECL Western Blotting Substrate (Thermo Fisher Scientific, 32106) and exposed to the X-ray film (AGFA, CP-GU M) or acquired with the iBright CL1500 Imaging System (Invitrogen).

### Microscopy

Cell images were taken using the Leica DM RXA2 microscope equipped with a mCherry/GFP/DAPI (4',6-diamidino-2-phenylindole) filter set and acquired by the MetaMorph imaging software. Cells were grown in liquid YPD medium at 30°C for ~18 h or on YPD plates at 30°C for ~2 days and then collected and resuspended in phosphate-buffered saline (PBS). One microliter of the cell suspension was applied to a glass microscope slide and visualized under a microscope (100× magnification). Differential interference contrast optics were used for standard cell morphology. Images of colonies were captured using the Leica MZ16 fluorescence stereomicroscope or the Bio-Rad Gel Doc XR + gel imaging system. Cells were spotted or spread onto YPD plates and grown at 30°C for 2–5 days to form colonies.

### RNA preparation

For RNA-Seq, overnight cultures were washed three times with PBS and pelleted for total RNA extraction using the hot phenol method. Briefly, cell pellets were resuspended in TES solution (10 mM Tris pH 7.5, 10 mM EDTA pH 8.0, 0.5% SDS) with acidic phenol (Sigma-Aldrich, P1037) and incubated at 65°C for 20 min. After centrifugation, the aqueous layer was transferred to a fresh tube and mixed with chloroform (Sigma-Aldrich, C2432) by vortexing. Then, the aqueous phase was collected by centrifugation and transferred to a clean tube. To precipitate RNA, sodium acetate (3 M pH 5.3) (1/10 of the volume of the aqueous phase) and an equal volume of ice-cold 100% ethanol were added to the above aqueous phase, gently mixed by inversion. Precipitated RNA was washed with ice-cold 70% ethanol and air-dried. To remove genomic DNA contamination, total RNA was treated with TURBO DNase (Thermo Fisher Scientific, AM2238). The DNA-free RNA was then precipitated, washed, and air-dried. The RNA pellet was dissolved in nuclease-free water and stored at −80°C until use.

For qPCR analysis of gene expression, overnight cultures were harvested for RNA isolation using the RNeasy Mini Kit (Qiagen, 74104) and the RNase-Free DNase Set (Qiagen, 79254) according to the manufacturer’s instructions.

### RNA-Seq analysis

Total RNA was extracted as described above. Illumina library preparation and sequencing were then performed by Singapore Immunology Network on samples from three independent experiments following standard operating procedures. We obtained ~21 million read pairs for each sample. Raw sequencing data quality was analyzed using FastQC (version 0.11.4; http://www.bioinformatics.babraham.ac.uk/projects/fastqc) and processed by Trim Galore (version 0.6.0; http://www.bioinformatics.babraham.ac.uk/projects/trim_galore/) to remove adapters and low-quality reads. Clean reads were aligned to the *C. auris* B8441 reference genome (GenBank assembly accession no. GCA_002759435.2) using the HISAT2 (version 2.1.0) software (68). Read counts mapped to individual transcripts were then normalized to log_2_ transformed transcripts per kilobase million, followed by quantile normalization. Significant differential expression was detected using the DESeq2 package (version 1.22.1) (69). Genes with a *P*-value <0.05 and a log_2_ fold change value ≥1 or ≤−1 were considered to represent DEGs. The GO analysis of DEGs was performed using the Gene Ontology Term Finder of *Candida* Genome Database (http://www.candidagenome.org/cgi-bin/GO/goTermFinder). All analyses were performed using R3.6.1 (https://www.r-project.org/). The R package ggplot2 (version 3.3.5; https://ggplot2.tidyverse.org/), VennDiagram (version 1.6.20; https://CRAN.R-project.org/package=VennDiagram), and ggpubr (version 0.4.0; https://rpkgs.datanovia.com/ggpubr/) were used to plot the figures in Fig. 2A through D.

### Quantitative PCR

Total RNA extracted from samples collected from three independent experiments was reverse-transcribed to cDNA using the LunaScript RT SuperMix Kit (NEB, E3010L). qPCR was performed using the Luna Universal qPCR Master Mix (NEB, M3003E) on a Bio-Rad CFX96 real-time system using the following cycling parameters: 95°C for 3 min, 95°C for 15 s, and 60°C for 30 s for 40 cycles. Normalization was done against the expression level of *GPD1*. Primer sequences for qPCR analysis are listed in Table S2.

### R6G and NR efflux assay

Overnight cultures were pelleted, washed twice with PBS, and diluted to 1 × 10^7^ cells/mL in PBS. Cells were mixed with R6G (Sigma-Aldrich, 252433) or NR (Sigma-Aldrich, 72485) at a final concentration of 10 µM or 7.5 µM, respectively. Cells were incubated at 30°C with shaking for 2 h, washed twice with PBS, and then resuspended in PBS. For measurement of R6G efflux, cells were removed by centrifugation and the supernatants were transferred to a 96-well flat-bottom microplate (Thermo Fisher Scientific, 167008). The fluorescence was measured using an excitation wavelength of 515 nm and an emission wavelength of 555 nm. For measurement of NR efflux, cells suspensions were transferred to a 96-well black flat-bottom microplate (Greiner Bio-One, 655900). The fluorescence was measured using an excitation wavelength of 530 nm and an emission wavelength of 635 nm.

### DNA pull-down assay

DNA pull-down experiments were performed as described previously (70). The biotinylated probes were PCR-amplified from *C. auris* genomic DNA using primers with a 5´-TEG-biotin modification and concentrated using Amicon Ultra-0.5 centrifugal filter (Merck Millipore, UFC503096). The DNA probes were affixed to the streptavidin-conjugated Dynabeads (Thermo Fisher Scientific, 65002) at room temperature with agitation for 30 min, followed by three washes with the 2× B/W buffer (10 mM Tris pH 7.5, 1 mM EDTA, 2 M NaCl) and once with the BS/THES buffer (22 mM Tris pH 7.5, 4.4 mM EDTA, 8.9% sucrose, 62 mM NaCl, 10 mM HEPES, 5 mM CaCl_2_, 50 mM KCl, 12% glycerol, 1× complete protease inhibitor cocktail). The DNA-bound beads were incubated with the prepared cell lysates at room temperature with agitation for 1 h, followed by five washes with the BS/THES buffer. Bead-bound proteins were eluted by boiling in 2× Laemmli sample buffer. Eluted proteins were separated by 10% SDS-PAGE and then immunoblotted with anti-Myc antibody. Primer sequences used for PCR amplification of the biotinylated DNA probes are listed in Table S2.

### Recombinant protein purification and gel mobility shift assay

The coding sequence of *RPN4* was amplified by PCR and cloned into pET28a vector to generate the expression plasmid. The resulting construct was transformed into the *E. coli* strain BL21. Cells were grown to an OD_600_ of ~0.8 and expression was induced with 0.2 mM isopropyl-β-D-thiogalactoside (GOLDBIO, I2481C100). After ~3 h induction, cells were pelleted and lysed in lysis buffer (50 mM sodium phosphate buffer pH 7.5 containing 300 mM NaCl, 2% glycerol, 1% NP-40, 4 mM β-mercaptoethanol, 1× complete protease inhibitor cocktail) by sonication. The supernatants of cell lysates containing His-tagged Rpn4 were purified by TALON Metal Affinity Resins (TaKaRa, 635504) and eluted by imidazole in PBS. Amicon Ultra-15 centrifugal filters (Merck Millipore, UFC901024) were used to exchange buffer and concentrate the protein.

Gel mobility shift assay was carried out using the fluorescence-based Electrophoretic Mobility Shift Assay (EMSA) Kit (Thermo Fisher Scientific, E33075) according to the manufacturer’s instruction. The DNA Probes were PCR-amplified from *C. auris* genomic DNA and purified from agarose gel using the E.Z.N.A. Gel Extraction Kit. Binding reaction mixtures were incubated for 1 h at room temperature. Samples were loaded onto a 6% nondenaturing polyacrylamide gel and run for 50 min at 30 mA in pre-chilled 1× Tris-borate-EDTA (TBE) buffer. The gel was subsequently stained in 1× SYBR Green EMSA stain with continuous, gentle agitation for 50 min, protected from light. Imaging was conducted on the iBright CL1500 Imaging System using a filter set appropriate for visualizing fluorescein (fluorescein isothiocyanate [FITC]). Primer sequences used for PCR amplification of the DNA Probes are listed in Table S2.

### Y1H and Y2H assays

The Y1H assay was carried out using the Matchmaker Gold Yeast One-Hybrid Library Screening System (TaKaRa, 630491). The WT and the modified *CDR1* promoter fragments were cloned into the pAbAi vector to create bait constructs. The linearized bait plasmids were then integrated into the Y1H Gold yeast strain and selected on leucine-deficient SD plates (SD/-Leu) to generate a bait-specific reporter strain. Subsequently, the coding sequences of *RPN4* was cloned into the prey vector pGADT7 and transformed into the Y1HGold bait reporter yeast strain constructed above. The activation of the AbA^r^ reporter gene on the bait vector was assessed on SD plate containing the aureobasidin A (SD/-Leu/+AbA) to detect protein-DNA interaction.

The Matchmaker system 3 (Clontech, PT3247-1) was used for Y2H analysis. The coding sequences of *UBR2* and *MUB1* were cloned into the prey vector pGADT7 and the bait vector pGBKT7, respectively. The resulting plasmids were co-transformed into the AH109 strain, and transformants were selected on the double dropout medium (SD/-Leu/-Trp). The activation of the *HIS3* and *ADE2* reporter genes was assessed on the quadruple dropout medium (SD/-Ade/-His/-Leu/-Trp) to detect protein-protein interaction.

### Variant identification

The genome sequence data of 304 globally distributed *C. auris* clinical isolates was obtained from the prior study (4). Raw sequencing data quality was analyzed using FastQC (version 0.11.4; http://www.bioinformatics.babraham.ac.uk/projects/fastqc) and processed by Trim Galore (version 0.6.0; http://www.bioinformatics.babraham.ac.uk/projects/trim_galore/) to remove adapters and low-quality reads. Clean reads were aligned to the *C. auris* B8441 reference genome (GenBank assembly accession no. GCA_002759435.2) using the BWA (version 0.7.15) software (71). Variants were then identified and filtered by GATK (version 3.5) (72) using the following parameters, “QD < 2.0/FS > 60.0/MQ < 40.0” for SNPs and “QD < 2.0/FS > 200.0” for indels. ANNOVAR (73) was used to perform gene-based annotation. The non-synonymous variants associated with fluconazole resistance were determined using Fisher’s exact test with odds ratio >1 and adjusted *P*-value <0.05.

### Structure prediction

The Ubr2/Mub1/Rpn4 complex structure was predicted by AlphaFold2-Multimer (52) through a subscription to the Google Colab (https://colab.research.google.com/github/sokrypton/ColabFold/blob/main/AlphaFold2.ipynb) following the provided guidelines. PyMOL (The PyMOL Molecular Graphics System, Version 2.5, Schrödinger, LLC) was used to visualize and align protein structures.

### Statistics and reproducibility

All experiments were independently repeated at least three times with similar results. Data shown in the figures represent the technical replicates from a single biological replicate or the average of three biological replicates. Error bars represent the standard deviation. Statistical analyses were performed using two-tailed unpaired Student’s *t*-test, two-way analysis of variance, followed by a multiple comparison test with the Geisser-Greenhouse correction using GraphPad Prism 8, unless otherwise stated.

## Data Availability

The RNA-Seq data are deposited under GEO accession number GSE218140.

## SUPPLEMENTAL MATERIAL

Data S1 mbio.02688-23-s0001.xlsx Differentially expressed genes in *ubr2*Δ and *mub1*Δ cells.

Data S2 mbio.02688-23-s0002.xlsx GO term enrichment analysis of the overlapped differentially expressed genes between *ubr2*Δ and *mub1*Δ cells.

Data S3 mbio.02688-23-s0003.xlsx Identification of mutations associated with fluconazole resistance among 304 globally distributed *C. auris* clinical isolates.

Supplemental materials mbio.02688-23-s0004.docx Supplemental figures and tables.

ASM does not own the copyrights to Supplemental Material that may be linked to, or accessed through, an article. The authors have granted ASM a non-exclusive, world-wide license to publish the Supplemental Material files. Please contact the corresponding author directly for reuse.
