# Supplementary material for: The transcription factor Rpn4 activates its own transcription and induces efflux pump expression to confer fluconazole resistance in Candida auris
Source: mBio. 2023 Nov 28;14(6):e02688-23. doi: 10.1128/mbio.02688-23 (PMC10746192; doi:10.1128/mbio.02688-23)
Supplement: Supplemental materials — Supplemental figures and tables. [file mbio.02688-23-s0004.docx]

**The transcription factor Rpn4 activates its own and efflux pumps’ expression to confer fluconazole resistance in *Candida auris***

Eve W. L. Chow^1#^, Yabing Song^2,3#^, Jinxin Chen^2,3^, Xiaoli Xu^1^, Jianbin Wang^2,4^, Kun Chen^5,6^, Jiaxin Gao^3*^ and Yue Wang^1,7*^

^1^ Infectious Diseases Labs, Agency for Science, Technology and Research, Singapore

^2^ School of Life Sciences, Tsinghua University, Beijing, China

^3^ State Key Laboratory of Mycology, Institute of Microbiology, Chinese Academy of Sciences, Beijing, China

^4^ Beijing Frontier Research Center for Biological Structure, Tsinghua University, Beijing, China

^5^ Translational Medical Center for Stem Cell Therapy, Institute for Regenerative Medicine, Shanghai East Hospital, School of Life Sciences and Technology, Tongji University, Shanghai, 200127, China

^6^ Shanghai Key Laboratory of Signaling and Disease Research, Frontier Science Center for Stem Cell Research, School of Life Sciences and Technology, Tongji University, Shanghai, 200092, China

^7^ Department of Biochemistry, Yong Loo Lin School of Medicine, National University of Singapore, Singapore

^#^ These authors contributed equally

^*^ Co-corresponding authors; gaojx@im.ac.cn and wang_yue@idlabs.a-star.edu.sg

**Supplementary Figures, Figure Legends, and Tables**

**
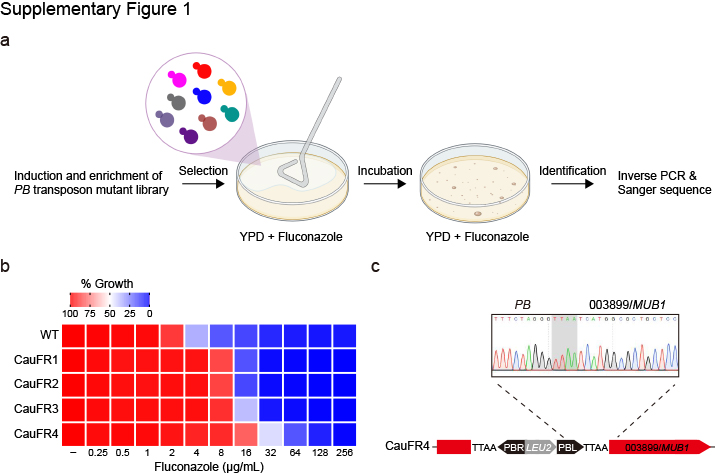
**

**Supplementary Fig. 1.** **Genetic screen for fluconazole-resistant mutants using *PB* mutagenesis system in *C. auris*.**

1. Illustration of the screening procedures.
2. Fluconazole susceptibility assays for the indicated mutants were performed as described in Fig. 1a. Fluconazole was applied as a 2-fold dilution series. Growth was measured and normalized to no-drug control. Data are representative of three technical replicates.
3. Genotype description and *PB* insertion site of the CauFR4 mutant.

**
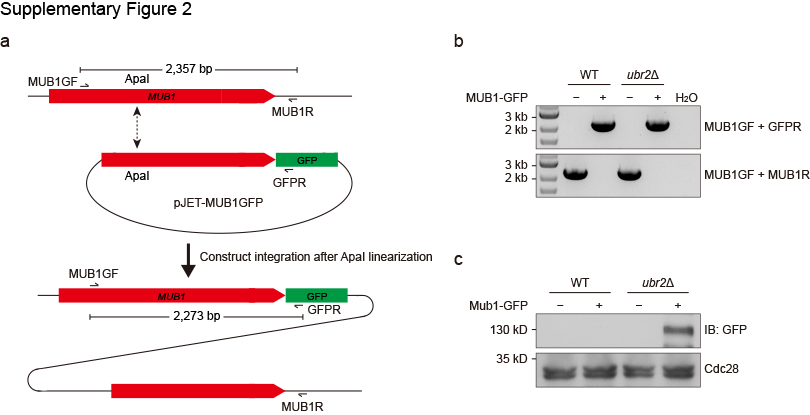
**

**Supplementary Fig. 2. Expression of Mub1-GFP under its native promoter by plasmid-based tagging.**

1. Illustration of plasmid integration used to fuse the C-terminus coding sequence of *MUB1* with the coding region of GFP. A Plasmid expressing Mub1-GFP, pJET-MUB1GFP, was linearized at the *Apa*I restriction site and integrated into the *C. auris* genome. Arrows indicate primers used for genotyping. MUB1GF + GFPR, spanning the *MUB1-GFP* junction, shows amplification only from strains expressing Mub1-GFP. MUB1GF + MUB1R, spanning the *MUB1* native locus, shows amplification only from strains without GFP tagging.

(b) PCR-based genotyping of WT and *ubr2*Δ cells containing Mub1-GFP using primers shown in panel (a). WT and *ubr2*Δ cells without GFP tagging were included as controls.

(c) The protein level of endogenously expressed Mub1-GFP in WT and *ubr2*Δ cells. WT and *ubr2*Δ cells without GFP tagging were included as controls. Cdc28 served as loading control.

**
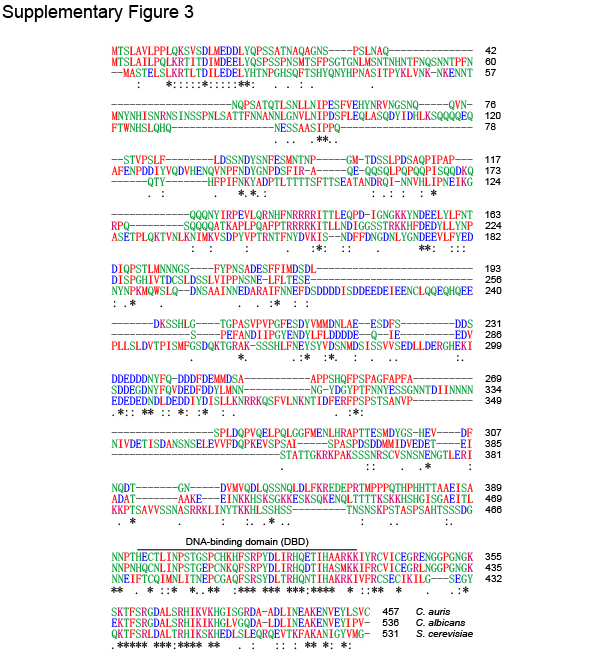
**

**Supplementary Fig. 3. Alignment of the Rpn4 protein sequences among *C. auris*, *C. albicans*, and *S. cerevisiae*.** Multiple sequence alignment was performed using Clustal Omega. The DNA-binding domain (DBD) is indicated above the sequences.

**
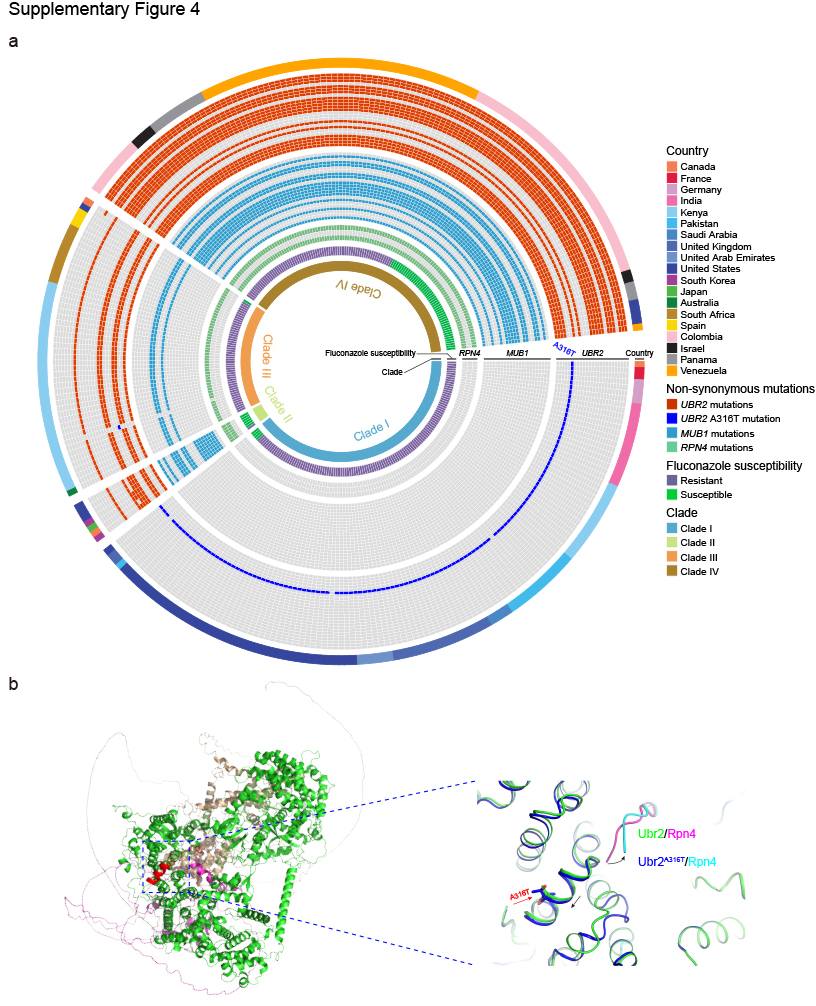
**

**Supplementary Fig. 4. Identification of mutations in *UBR2*, *MUB1*, and *RPN4*** **from whole-genome sequences of *C. auris* clinical isolates.**

1. The distribution of *UBR2*, *MUB1*, and *RPN4* mutations in four major clades. Country, non-synonymous polymorphism, susceptibility to fluconazole, and clade are color labeled as indicated.
2. Protein complex prediction with AlphaFold2-Multimer. Left: overall structure of the Ubr2/Mub1/Rpn4 trimer. Chains in green, wheat, and fuchsia show the structure of Ubr2, Mub1, and Rpn4, respectively. A possible interface formed by Ubr2 Ala^316^-associated α-helix and nearby Rpn4 loop is labeled in red and highlighted in the blue dashed line box. Right: close-up view of the UBR2^A316T^ mutation’s effect on this possible interface. Chains in green and fuchsia show the interface of Ubr2 and Rpn4. Chains in blue and cyan exhibit the interface formed by Ubr2^A316T^ and Rpn4. The red arrow indicates the UBR2^A316T^ mutation. The black arrows indicate the potential shifts caused by the UBR2^A316T^ mutation.

**Supplementary Table 1. *C. auris* strains used in this study.**

| Strains | Genotype | Clade |  | Source |
| --- | --- | --- | --- | --- |
| CBS10913 | WT, Japan | II |  | **From Westerdijk Fungal Biodiversity Institute** |
| BJCA001 | WT, China | I |  | **From Huang Guanghua Lab** |
| CauW49 | CBS10913 *leu2*Δ:*FRT* | II |  | This study |
| CauFR4 | CBS10913 *leu2*Δ:*FRT P_Tet-On_-CaPBase:SAT1*  *003899:PB[LEU2]* (TTAA, n.t. 650-653 within the ORF of *003899*, reverse strand) | II |  | This study |
| CauW50 | CBS10913 *leu2*Δ:*FRT mub1*Δ:*LEU2* | II |  | This study |
| CauW51 | CBS10913 *leu2*Δ:*FRT mub1*Δ:*LEU2 MUB1:SAT1* | II |  | This study |
| CauW52 | CBS10913 *leu2*Δ:*FRT mub1*Δ:*LEU2 UBR2-Myc:SAT1* | II |  | This study |
| CauW53 | CBS10913 *leu2*Δ:*FRT ubr2*Δ:*LEU2 MUB1-GFP:SAT1* | II |  | This study |
| CauW54 | CBS10913 *leu2*Δ:*FRT ubr2*Δ:*LEU2* | II |  | This study |
| CauW55 | CBS10913 *leu2*Δ:*FRT ubr2*Δ:*LEU2 UBR2:SAT1* | II |  | This study |
| CauW56 | BJCA001 *ubr2*Δ:*SAT1* | I |  | This study |
| CauW57 | BJCA001 *mub1*Δ:*SAT1* | I |  | This study |
| CauW58 | CBS10913 *RPN4-Myc:SAT1* | II |  | This study |
| CauW59 | CBS10913 *leu2*Δ:*FRT ubr2*Δ:*LEU2 RPN4-Myc:SAT1* | II |  | This study |
| CauW60 | CBS10913 *leu2*Δ:*FRT mub1*Δ:*LEU2 RPN4-Myc:SAT1* | II |  | This study |
| CauW61 | CBS10913 *leu2*Δ:*FRT* *GFP-RPN4:SAT1 NAB2-mCherry:LEU2* | II |  | This study |
| CauW62 | CBS10913 *leu2*Δ:*FRT ubr2*Δ:*HYG GFP-RPN4:SAT1 NAB2-mCherry:LEU2* | II |  | This study |
| CauW63 | CBS10913 *leu2*Δ:*FRT mub1*Δ:*HYG GFP-RPN4:SAT1 NAB2-mCherry:LEU2* | II |  | This study |
| CauW64 | CBS10913 *rpn4*Δ:*SAT1* | II |  | This study |
| CauW65 | CBS10913 *leu2*Δ:*FRT ubr2*Δ:*LEU2 rpn4*Δ:*SAT1* | II |  | This study |
| CauW66 | CBS10913 *leu2*Δ:*FRT mub1*Δ:*LEU2 rpn4*Δ:*SAT1* | II |  | This study |
| CauW67 | CBS10913 *leu2*Δ:*FRT snq21*Δ:*LEU2* | II |  | This study |
| CauW68 | CBS10913 *leu2*Δ:*FRT snq22*Δ:*LEU2* | II |  | This study |
| CauW69 | CBS10913 *mdr1*Δ:*SAT1* | II |  | This study |
| CauW70 | CBS10913 *cdr1*Δ:*SAT1* | II |  | This study |
| CauW71 | CBS10913 *leu2*Δ:*FRT cdr1*Δ:*LEU2* *snq21*Δ:*FRT* | II |  | This study |
| CauW72 | CBS10913 *leu2*Δ:*FRT cdr1*Δ:*LEU2* *snq22*Δ:*SAT1-FLP* | II |  | This study |
| CauW73 | CBS10913 *cdr1*Δ:*SAT1* *mdr1*Δ:*HYG* | II |  | This study |
| CauW74 | CBS10913 *leu2*Δ:*FRT cdr1*Δ:*LEU2* *snq21*Δ:*FRT snq22*Δ:*FRT* *mdr1*Δ:*SAT1* | II |  | This study |
| CauW75 | CBS10913 *leu2*Δ:*FRT ubr2*Δ:*HYG* *snq21*Δ:*LEU2* | II |  | This study |
| CauW76 | CBS10913 *leu2*Δ:*FRT ubr2*Δ:*HYG* *snq22*Δ:*LEU2* | II |  | This study |
| CauW77 | CBS10913 *leu2*Δ:*FRT ubr2*Δ:*LEU2* *mdr1*Δ:*SAT1* | II |  | This study |
| CauW78 | CBS10913 *leu2*Δ:*FRT ubr2*Δ:*LEU2* *cdr1*Δ:*SAT1* | II |  | This study |
| CauW79 | CBS10913 *leu2*Δ:*FRT ubr2*Δ:*HYG* *cdr1*Δ:*LEU2* *snq21*Δ:*FRT* | II |  | This study |
| CauW80 | CBS10913 *leu2*Δ:*FRT ubr2*Δ:*HYG* *cdr1*Δ:*LEU2* *snq22*Δ:*SAT1-FLP* | II |  | This study |
| CauW81 | CBS10913 *leu2*Δ:*FRT ubr2*Δ:*LEU2* *cdr1*Δ:*SAT1* *mdr1*Δ:*HYG* | II |  | This study |
| CauW82 | CBS10913 *leu2*Δ:*FRT ubr2*Δ:*HYG* *cdr1*Δ:*LEU2* *snq21*Δ:*FRT snq22*Δ:*FRT* *mdr1*Δ:*SAT1* | II |  | This study |
| CauW83 | CBS10913 *leu2*Δ:*FRT mub1*Δ:*LEU2* *snq21*Δ:*SAT1-FLP* | II |  | This study |
| CauW84 | CBS10913 *leu2*Δ:*FRT mub1*Δ:*LEU2* *snq22*Δ:*SAT1-FLP* | II |  | This study |
| CauW85 | CBS10913 *leu2*Δ:*FRT mub1*Δ:*LEU2* *mdr1*Δ:*SAT1* | II |  | This study |
| CauW86 | CBS10913 *leu2*Δ:*FRT mub1*Δ:*LEU2* *cdr1*Δ:*SAT1* | II |  | This study |
| CauW87 | CBS10913 *leu2*Δ:*FRT mub1*Δ:*HYG* *cdr1*Δ:*LEU2* *snq21*Δ:*FRT* | II |  | This study |
| CauW88 | CBS10913 *leu2*Δ:*FRT mub1*Δ:*HYG* *cdr1*Δ:*LEU2* *snq22*Δ:*SAT1-FLP* | II |  | This study |
| CauW89 | CBS10913 *leu2*Δ:*FRT mub1*Δ:*LEU2* *cdr1*Δ:*SAT1* *mdr1*Δ:*HYG* | II |  | This study |
| CauW90 | CBS10913 *leu2*Δ:*FRT mub1*Δ:*HYG* *cdr1*Δ:*LEU2* *snq21*Δ:*FRT snq22*Δ:*FRT* *mdr1*Δ:*SAT1* | II |  | This study |
| CauW91 | CBS10913 *P_PRN4_-*tdTomato:*HYG* | II |  | This study |
| CauW92 | CBS10913 *rpn4*Δ:*SAT1 P_PRN4_-*tdTomato:*HYG* | II |  | This study |
| CauW93 | CBS10913 *leu2*Δ:*FRT ubr2*Δ:*LEU2* *P_PRN4_-*tdTomato:*HYG* | II |  | This study |
| CauW94 | CBS10913 *leu2*Δ:*FRT ubr2*Δ:*LEU2* *rpn4*Δ:*SAT1* *P_PRN4_-*tdTomato:*HYG* | II |  | This study |
| CauW95 | CBS10913 *leu2*Δ:*FRT mub1*Δ:*LEU2* *P_PRN4_-*tdTomato:*HYG* | II |  | This study |
| CauW96 | CBS10913 *leu2*Δ:*FRT mub1*Δ:*LEU2* *rpn4*Δ:*SAT1* *P_PRN4_-*tdTomato:*HYG* | II |  | This study |
| CauW97 | CBS10913 *rpn4*Δ:*SAT1* *P_PRN4_-RPN4:HYG* | II |  | This study |
| CauW98 | CBS10913 *rpn4*Δ:*SAT1* *P_PRN4d_-RPN4:HYG* | II |  | This study |
| CauW99 | CBS10913 *rpn4*Δ:*SAT1* *P_PRN4m_-RPN4:HYG* | II |  | This study |
| CauW100 | CBS10913 *leu2*Δ:*FRT ubr2*Δ:*LEU2* *rpn4*Δ:*SAT1* *P_PRN4_-RPN4:HYG* | II |  | This study |
| CauW101 | CBS10913 *leu2*Δ:*FRT ubr2*Δ:*LEU2* *rpn4*Δ:*SAT1* *P_PRN4d_-RPN4:HYG* | II |  | This study |
| CauW102 | CBS10913 *leu2*Δ:*FRT ubr2*Δ:*LEU2* *rpn4*Δ:*SAT1* *P_PRN4m_-RPN4:HYG* | II |  | This study |
| CauW103 | CBS10913 *leu2*Δ:*FRT mub1*Δ:*LEU2* *rpn4*Δ:*SAT1* *P_PRN4_-RPN4:HYG* | II |  | This study |
| CauW104 | CBS10913 *leu2*Δ:*FRT mub1*Δ:*LEU2* *rpn4*Δ:*SAT1* *P_PRN4d_-RPN4:HYG* | II |  | This study |
| CauW105 | CBS10913 *leu2*Δ:*FRT mub1*Δ:*LEU2* *rpn4*Δ:*SAT1* *P_PRN4m_-RPN4:HYG* | II |  | This study |
| CauW106 | CBS10913 *tac1a*Δ:*SAT1-FLP* | II |  | This study |
| CauW107 | CBS10913 *tac1b*Δ:*SAT1-FLP* | II |  | This study |
| CauW108 | CBS10913 *tac1a*Δ:*FRT* *tac1b*Δ:*SAT1-FLP* | II |  | This study |
| CauW109 | CBS10913 *mrr1a*Δ:*SAT1-FLP* | II |  | This study |
| CauW110 | CBS10913 *mrr1b*Δ:*SAT1-FLP* | II |  | This study |
| CauW111 | CBS10913 *mrr1c*Δ:*SAT1-FLP* | II |  | This study |
| CauW112 | CBS10913 *mrr1a*Δ:*FRT* *mrr1b*Δ:*SAT1-FLP* | II |  | This study |
| CauW113 | CBS10913 *mrr1b*Δ:*SAT1-FLP* *mrr1c*Δ:*FRT* | II |  | This study |
| CauW114 | CBS10913 *mrr1a*Δ:*FRT* *mrr1c*Δ:*SAT1-FLP* | II |  | This study |
| CauW115 | CBS10913 *mrr1a*Δ:*SAT1-FLP* *mrr1b*Δ:*FRT* *mrr1c*Δ:*FRT* | II |  | This study |
| CauW116 | CBS10913 *tac1a*Δ:*FRT* *tac1b*Δ:*FRT* *mrr1a*Δ:*FRT* *mrr1b*Δ:*SAT1-FLP* *mrr1c*Δ:*FRT* | II |  | This study |
| CauW117 | CBS10913 *ubr2*Δ:*HYG* *tac1a*Δ:*FRT* | II |  | This study |
| CauW118 | CBS10913 *ubr2*Δ:*HYG* *tac1b*Δ:*SAT1-FLP* | II |  | This study |
| CauW119 | CBS10913 *ubr2*Δ:*HYG* *tac1a*Δ:*FRT* *tac1b*Δ:*SAT1-FLP* | II |  | This study |
| CauW120 | CBS10913 *ubr2*Δ:*HYG* *mrr1a*Δ:*SAT1-FLP* | II |  | This study |
| CauW121 | CBS10913 *ubr2*Δ:*HYG* *mrr1b*Δ:*SAT1-FLP* | II |  | This study |
| CauW122 | CBS10913 *ubr2*Δ:*HYG* *mrr1c*Δ:*SAT1-FLP* | II |  | This study |
| CauW123 | CBS10913 *ubr2*Δ:*HYG* *mrr1a*Δ:*FRT* *mrr1b*Δ:*SAT1-FLP* | II |  | This study |
| CauW124 | CBS10913 *ubr2*Δ:*HYG* *mrr1b*Δ:*SAT1-FLP* *mrr1c*Δ:*FRT* | II |  | This study |
| CauW125 | CBS10913 *ubr2*Δ:*HYG* *mrr1a*Δ:*FRT* *mrr1c*Δ:*SAT1-FLP* | II |  | This study |
| CauW126 | CBS10913 *ubr2*Δ:*HYG* *mrr1a*Δ:*SAT1-FLP* *mrr1b*Δ:*FRT* *mrr1c*Δ:*FRT* | II |  | This study |
| CauW127 | CBS10913 *ubr2*Δ:*HYG* *tac1a*Δ:*FRT* *tac1b*Δ:*FRT* *mrr1a*Δ:*FRT* *mrr1b*Δ:*SAT1-FLP* *mrr1c*Δ:*FRT* | II |  | This study |
| CauW128 | CBS10913 *mub1*Δ:*HYG* *tac1a*Δ:*FRT* | II |  | This study |
| CauW129 | CBS10913 *mub1*Δ:*HYG* *tac1b*Δ:*SAT1-FLP* | II |  | This study |
| CauW130 | CBS10913 *mub1*Δ:*HYG* *tac1a*Δ:*FRT* *tac1b*Δ:*SAT1-FLP* | II |  | This study |
| CauW131 | CBS10913 *mub1*Δ:*HYG* *mrr1a*Δ:*SAT1-FLP* | II |  | This study |
| CauW132 | CBS10913 *mub1*Δ:*HYG* *mrr1b*Δ:*SAT1-FLP* | II |  | This study |
| CauW133 | CBS10913 *mub1*Δ:*HYG* *mrr1c*Δ:*SAT1-FLP* | II |  | This study |
| CauW134 | CBS10913 *mub1*Δ:*HYG* *mrr1a*Δ:*FRT* *mrr1b*Δ:*SAT1-FLP* | II |  | This study |
| CauW135 | CBS10913 *mub1*Δ:*HYG* *mrr1b*Δ:*SAT1-FLP* *mrr1c*Δ:*FRT* | II |  | This study |
| CauW136 | CBS10913 *mub1*Δ:*HYG* *mrr1a*Δ:*FRT* *mrr1c*Δ:*SAT1-FLP* | II |  | This study |
| CauW137 | CBS10913 *mub1*Δ:*HYG* *mrr1a*Δ:*SAT1-FLP* *mrr1b*Δ:*FRT* *mrr1c*Δ:*FRT* | II |  | This study |
| CauW138 | CBS10913 *mub1*Δ:*HYG* *tac1a*Δ:*FRT* *tac1b*Δ:*FRT* *mrr1a*Δ:*FRT* *mrr1b*Δ:*SAT1-FLP* *mrr1c*Δ:*FRT* | II |  | This study |
| CauW139 | CBS10913 *leu2*Δ:*FRT cdr1*Δ:*LEU2* *P_CDR1_-CDR1:HYG* | II |  | This study |
| CauW140 | CBS10913 *leu2*Δ:*FRT cdr1*Δ:*LEU2* *P_CDR1d_-CDR1:HRG* | II |  | This study |
| CauW141 | CBS10913 *leu2*Δ:*FRT cdr1*Δ:*LEU2* *P_CDR1m_-CDR1:HYG* | II |  | This study |
| CauW142 | CBS10913 *leu2*Δ:*FRT ubr2*Δ:*SAT1* *cdr1*Δ:*LEU2* *P_CDR1_-CDR1:HYG* | II |  | This study |
| CauW143 | CBS10913 *leu2*Δ:*FRT ubr2*Δ:*SAT1* *cdr1*Δ:*LEU2* *P_CDR1d_-CDR1:HYG* | II |  | This study |
| CauW144 | CBS10913 *leu2*Δ:*FRT ubr2*Δ:*SAT1* *cdr1*Δ:*LEU2* *P_CDR1m_-CDR1:HYG* | II |  | This study |
| CauW145 | CBS10913 *leu2*Δ:*FRT mub1*Δ:*SAT1* *cdr1*Δ:*LEU2* *P_CDR1_-CDR1:HYG* | II |  | This study |
| CauW146 | CBS10913 *leu2*Δ:*FRT mub1*Δ:*SAT1* *cdr1*Δ:*LEU2* *P_CDR1d_-CDR1:HYG* | II |  | This study |
| CauW147 | CBS10913 *leu2*Δ:*FRT mub1*Δ:*SAT1* *cdr1*Δ:*LEU2* *P_CDR1m_-CDR1:HYG* | II |  | This study |
| CauW148 | CBS10913 *leu2*Δ:*FRT ubr2*Δ:*LEU2 UBR2*^A316T^:*SAT1* | II |  | This study |
| CauW149 | CBS10913 *leu2*Δ:*FRT ubr2*Δ:*LEU2 UBR2*^A316T^:*SAT1 rpn4*Δ: *HYG* | II |  | This study |
| CauW150 | CBS10913 *leu2*Δ:*FRT ubr2*Δ:*LEU2 UBR2:SAT1 RPN4-Myc: HYG* | II |  | This study |
| CauW151 | CBS10913 *leu2*Δ:*FRT ubr2*Δ:*LEU2 UBR2*^A316T^:*SAT1 RPN4-Myc: HYG* | II |  | This study |

**Supplementary Table 2. Primers used in this study.**

| Primer Name | Sequence (5’ to 3’) | Purpose |
| --- | --- | --- |
| SNQ21SFuF | ACATGCATGCGACTAAGAGGTCCGCATT | *SAT1* flipping-based gene disruption |
| SNQ21SFuR | CGGGATCCGAAGTTCCTATTCTCTAGAAAGTATAGGAACTTCAGCCAATGCAAGAGGAT |  |
| SNQ21SFdF | ATAAGAATGCGGCCGCAAGGAGATGGCTTACGTAT |  |
| SNQ21SFdR | TCCCCGCGGTCCGATCCTGATCCGAAAT |  |
| SNQ22SFuF | ACATGCATGCCAGAAGCGAATCCAGTGAT |  |
| SNQ22SFuR | CGGGATCCGAAGTTCCTATTCTCTAGAAAGTATAGGAACTTCCTGAAGAAACTCTCTT |  |
| SNQ22SFdF | ATAAGAATGCGGCCGCTGATGCACATGTGCGAA |  |
| SNQ22SFdR | TCCCCGCGGACTGTTGGTGGTACCGTTA |  |
| TAC1aSFuF | ACATGCATGCTGAACGGCTTCATA |  |
| TAC1aSFuR | CGGGATCCGAAGTTCCTATTCTCTAGAAAGTATAGGAACTTCCACTCACAGTTCGTGAA |  |
| TAC1aSFdF | ATAAGAATGCGGCCGCCACGCTGCTTATGTTCAA |  |
| TAC1aSFdR | TCCCCGCGGATGAGCGACAGCCGGCAA |  |
| TAC1bSFuF | ACATGCATGCTAGGCCAGGAAGTGACCTA |  |
| TAC1bSFuR | CGGGATCCGAAGTTCCTATTCTCTAGAAAGTATAGGAACTTCGCTTCTTGAGATTCGAA |  |
| TAC1bSFdF | ATAAGAATGCGGCCGCGCAAAGTGACGACAGGAA |  |
| TAC1bSFdR | TCCCCGCGGTGCGCAGTTGGCTATGAA |  |
| MRR1aSFuF | ACATGCATGCAATCCGGAGATGTGCAA |  |
| MRR1aSFuR | CGGGATCCGAAGTTCCTATTCTCTAGAAAGTATAGGAACTTCCTGCCAGATCTTTCGAA |  |
| MRR1aSFdF | ATAAGAATGCGGCCGCGGCGTCGTATTAGGAAA |  |
| MRR1aSFdR | TCCCCGCGGCAGGTCACTTGAACAA |  |
| MRR1bSFuF | ACATGCATGCGGACCTTGAGCAAGACAT |  |
| MRR1bSFuR | CGGGATCCGAAGTTCCTATTCTCTAGAAAGTATAGGAACTTCTGCACCATGACTAGTTA |  |
| MRR1bSFdF | ATAAGAATGCGGCCGCTCTCGACTGAGGCGATT |  |
| MRR1bSFdR | TCCCCGCGGCTGGCTGTTAGTGATAAAGCT |  |
| MRR1cSFuF | ACATGCATGCTCATCGAGAGATCCTCTT |  |
| MRR1cSFuR | CGGGATCCGAAGTTCCTATTCTCTAGAAAGTATAGGAACTTCTAGCGTTTGCAGGGTCAA |  |
| MRR1cSFdF | ATAAGAATGCGGCCGCCTCATCAAGTTGGGACAA |  |
| MRR1cSFdR | TCCCCGCGGCTGGAGGGATATGTCCATA |  |
| MUB1uF | GTCACTTGCGCGTGCAACCCAATA | Overlapping  PCR-based gene disruption |
| MUB1dR | TGGGAAAGCCTCTCCTTTCTCAGA |  |
| MUB1LuR | CTGATCTCAATCCTCCACACTGACTATGCC |  |
| MUB1LF | GGCATAGTCAGTGTGGAGGATTGAGATCAG |  |
| MUB1LR | TTATGCGTTGCATCAAAGATCTAATCGACT |  |
| MUB1LdF | AGTCGATTAGATCTTTGATGCAACGCATAA |  |
| MUB1SuR | TCTCTAGTTTTGACGCACACTGACTATGCC |  |
| MUB1SF | GGCATAGTCAGTGTGCGTCAAAACTAGAGA |  |
| MUB1SR | TTATGCGTTGCATCAGACCACCTTTGATTG |  |
| MUB1SdF | CAATCAAAGGTGGTCTGATGCAACGCATAA |  |
| MUB1HuR | AAGCTAAACAGATCTCACACTGACTATGCC |  |
| MUB1HF | GGCATAGTCAGTGTGAGATCTGTTTAGCTT |  |
| MUB1HR | TTATGCGTTGCATCAAGCTCGTTTTCGACA |  |
| MUB1HdF | TGTCGAAAACGAGCTTGATGCAACGCATAA |  |
| UBR2uF | GGGCTCCCATAGACAACTCCTCAAT |  |
| UBR2dR | GACCTGACCTTCAAGTTTTTGAAGA |  |
| UBR2LuR | CTGATCTCAATCCTCTTCTTGAGGAGACAA |  |
| UBR2LF | TTGTCTCCTCAAGAAGAGGATTGAGATCAG |  |
| UBR2LR | TTCTTGTAGTGAGTGAAGATCTAATCGACT |  |
| UBR2LdF | AGTCGATTAGATCTTCACTCACTACAAGAA |  |
| UBR2SuR | TCTCTAGTTTTGACGTTCTTGAGGAGACAA |  |
| UBR2SF | TTGTCTCCTCAAGAACGTCAAAACTAGAGA |  |
| UBR2SR | TTCTTGTAGTGAGTGGACCACCTTTGATTG |  |
| UBR2SdF | CAATCAAAGGTGGTCCACTCACTACAAGAA |  |
| UBR2HuR | AAGCTAAACAGATCTTTCTTGAGGAGACAA |  |
| UBR2HF | TTGTCTCCTCAAGAAAGATCTGTTTAGCTT |  |
| UBR2HR | TTCTTGTAGTGAGTGAGCTCGTTTTCGACA |  |
| UBR2HdF | TGTCGAAAACGAGCTCACTCACTACAAGAA |  |
| RPN4uF | CGGGAGCAACATGTTGGTGTTTTCA |  |
| RPN4dR | CTCCATCCTTCAGTACATCTCCGTA |  |
| RPN4SuR | TCTCTAGTTTTGACGATCAGATACGGACTT |  |
| RPN4SF | AAGTCCGTATCTGATCGTCAAAACTAGAGA |  |
| RPN4SR | AAGTCGGCAGCGTCCGACCACCTTTGATTG |  |
| RPN4SdF | CAATCAAAGGTGGTCGGACGCTGCCGACTT |  |
| RPN4HuR | AAGCTAAACAGATCTATCAGATACGGACTT |  |
| RPN4HF | AAGTCCGTATCTGATAGATCTGTTTAGCTT |  |
| RPN4HR | AAGTCGGCAGCGTCCAGCTCGTTTTCGACA |  |
| RPN4HdF | TGTCGAAAACGAGCTGGACGCTGCCGACTT |  |
| SNQ21uF | CTGCGCAAAGCACGATTTGACTAA |  |
| SNQ21dR | GAGACTCACTGGCCCAAAAATGACA |  |
| SNQ21LuR | CTGATCTCAATCCTCAGCCAATGCAAGAGG |  |
| SNQ21LF | CCTCTTGCATTGGCTGAGGATTGAGATCAG |  |
| SNQ21LR | ATACGTAAGCCATCTAAGATCTAATCGACT |  |
| SNQ21LdF | AGTCGATTAGATCTTAGATGGCTTACGTAT |  |
| SNQ22uF | CCTAGTCAGAAGCGAATCCAGTGAT |  |
| SNQ22dR | GCTGTGAACAAAGCAGGCTTGTT |  |
| SNQ22LuR | CTGATCTCAATCCTCCTGAAGAAACTCTCT |  |
| SNQ22LF | AGAGAGTTTCTTCAGGAGGATTGAGATCAG |  |
| SNQ22LR | AATCCAGGCATCAACAAGATCTAATCGACT |  |
| SNQ22LdF | AGTCGATTAGATCTTGTTGATGCCTGGATT |  |
| MDR1uF | GCTCCCACGGTATCTCGGAAACAA |  |
| MDR1dR | GGATTTGTTGAAAATCTCGTCTAT |  |
| MDR1SuR | TCTCTAGTTTTGACGGTGGAGATTGAAGAT |  |
| MDR1SF | ATCTTCAATCTCCACCGTCAAAACTAGAGA |  |
| MDR1SR | TAAGTGGACCAATCCGACCACCTTTGATTG |  |
| MDR1SdF | CAATCAAAGGTGGTCGGATTGGTCCACTTA |  |
| MDR1HuR | AAGCTAAACAGATCTGTGGAGATTGAAGAT |  |
| MDR1HF | ATCTTCAATCTCCACAGATCTGTTTAGCTT |  |
| MDR1HR | TAAGTGGACCAATCCAGCTCGTTTTCGACA |  |
| MDR1HdF | TGTCGAAAACGAGCTGGATTGGTCCACTTA |  |
| CDR1uF | GAATCCAAGGGTGGAATATGCGATT |  |
| CDR1dR | CATCAAATTGACCATGTTAGCAGCGTT |  |
| CDR1LuR | CTGATCTCAATCCTCAAAGTGGACTACATG |  |
| CDR1LF | CATGTAGTCCACTTTGAGGATTGAGATCAG |  |
| CDR1LR | ATAGCTTGACCGTGGAAGATCTAATCGACT |  |
| CDR1LdF | AGTCGATTAGATCTTCCACGGTCAAGCTAT |  |
| CDR1SuR | TCTCTAGTTTTGACGAAAGTGGACTACATG |  |
| CDR1SF | CATGTAGTCCACTTTCGTCAAAACTAGAGA |  |
| CDR1SR | ATAGCTTGACCGTGGGACCACCTTTGATTG |  |
| CDR1SdF | CAATCAAAGGTGGTCCCACGGTCAAGCTAT |  |
| SNQ21qF | TGGTTGCGTCGTTCCTCTAC | qPCR |
| SNQ21qR | TTGGCCCTCTGGTGGAGATA |  |
| SNQ22qF | TTCGGTGACATTGGCGATCA |  |
| SNQ22qR | CAAACCAGTCCCGATCCACA |  |
| MDR1qF | ACACTCCCCTTGACGCTTTT |  |
| MDR1qR | GAAAGCGGCGAAAACACCAT |  |
| CDR1qF | TCCCATTCCGTCGATGTTGG |  |
| CDR1qR | GTTGAGCACACACGGTTGAC |  |
| RPN4qF | CAAGCATTTTAGCCGGCCTT |  |
| RPN4qR | GACAAGGCATCACCTCTGGA |  |
| tdTomatoqF | AGGTGAAGGTGAAGGTAGACCA |  |
| tdTomatoqR | TCCCAAGCAAATGGTAATGGAC |  |
| GPD1qF | CTTGGCTGTCGGTTTTGTGG |  |
| GPD1qR | GTGAAAGTGCCAGACTCGGA |  |
| PRPN4F | GGTTCCAAGAGAGGGAAAACTGCAT | EMSA |
| PRPN4R | TTTTGTTGCTTCTCGAAGTGCTA |  |
| PCDR1F | GGAAGTGGAGACTCCAGTTGTT |  |
| PCDR1R | GGTTTCTCGGACATGGAGATGGAA |  |
| B_PRPN4F | B*GGTTCCAAGAGAGGGAAAACTGCAT | DNA pull-down |
| B_PRPN4R | B*TTTTGTTGCTTCTCGAAGTGCTA |  |
| B_RPN4F | B*ATGACTACTCGAACTTTGAAAGCAT |  |
| B_RPN4R | B*TCGGATTCCTCAGCGAGGTTGTCCAT |  |
| LEU2R | CGAGAGTCTCTTGATGTAGGCCTGGTC | Genotyping |
| SAT1R | TTGGGCAGTGCACACACTACTTAAT |  |
| HYGR | ATGTACGGGCGACAGTCACATCAT |  |
| MUB1F | TCATAGTGAGTATGGGATTCCTCCAA |  |
| MUB1kR | CTCTCACTTTGGCACTCAGCGAAA |  |
| UBR2F | AGGATGTCTCGCCGCTATCGCCA |  |
| UBR2kR | GCGTCACGAAAGTGGCGGATCT |  |
| RPN4F | GTTCGCGGCATTCGGAAAACGGA |  |
| RPN4kR | GGTCTGTGTGGCAGAGGGCTGATT |  |
| SNQ21F | GGGCGAGTCCAGGCCTGTCCAATT |  |
| SNQ21kR | GCTCGACTTCTTCACGACTAGCAT |  |
| SNQ22F | GCTTACATGCAGTATGTGCCGCTTA |  |
| SNQ22kR | CTGACTGCTTGACGTAGGTGTGAA |  |
| MDR1F | GGCTTGCGTGCTCAATGTCTACAGAA |  |
| MDR1kR | GGTGATTCCTCGGGGTATGTGAA |  |
| CDR1F | ACAACGTGCGGGAGTGAAGTATAGAA |  |
| CDR1kR | GCACCTCAGACATGTGGGACAA |  |
| TAC1aF | AGGGACTTCTTCGACGGCTTGAT |  |
| TAC1akR | GTCGATGGAGCTCTGGGACATTA |  |
| TAC1bF | GTTGCCTCCATGAAGCACATGAAA |  |
| TAC1bkR | GTAATGAAGCATCTGGTGGCCAA |  |
| MRR1aF | TACCTGCATCGGTGACACTCCAA |  |
| MRR1akR | GTTAGTGATGGAACCTGACGCATA |  |
| MRR1bF | GCCAAGGTCGAGCTGATCCGAA |  |
| MRR1bkR | GAGGCAACGGCTTCATCTTTTGC |  |
| MRR1cF | GTGACCAGGGCTCCATTGCAAT |  |
| MRR1ckR | CTCTTCGTAGGAAGCTTCTGTCTT |  |
| PENO1F | GCCACAGGGCATCTACACCCAATA |  |
| PJETR | CACTTTATGCTTCCGGCTCGTATAA |  |
| MUB1GF | ATGCATGGAGGCTCAAACAGTCAA |  |
| MUB1R | CGGTTGTCCATGGCCTACCACTT |  |
| GFPR | CCGTAAGTAGCATCACCTTCACCTT |  |

‘*’ denotes the biotinylated of the first nucleotide of B_PRPN4F, B_PRPN4R, B_RPN4F and B_RPN4R.
